# Totoro: Identifying Active Reactions During the Transient State for Metabolic Perturbations

**DOI:** 10.3389/fgene.2022.815476

**Published:** 2022-02-21

**Authors:** Mariana Galvão Ferrarini, Irene Ziska, Ricardo Andrade, Alice Julien-Laferrière, Louis Duchemin, Roberto Marcondes César, Arnaud Mary, Susana Vinga, Marie-France Sagot

**Affiliations:** ^1^ Laboratoire de Biométrie et Biologie Évolutive, UMR 5558, CNRS, Université de Lyon, Université Lyon 1, Villeurbanne, France; ^2^ Univ Lyon, INRAE, INSA-Lyon, BF2I, UMR 203, Villeurbanne, France; ^3^ INRIA Grenoble Rhône-Alpes, Villeurbanne, France; ^4^ Institute of Mathematics and Statistics (IME), University of São Paulo, São Paulo, Brazil; ^5^ Soladis GmBH, Basel, Switzerland; ^6^ INESC-ID, Instituto Superior Técnico, Universidade de Lisboa, Lisboa, Portugal

**Keywords:** metabolomics, metabolic networks, transient state, metabolic perturbation, omics integration

## Abstract

**Motivation:** The increasing availability of metabolomic data and their analysis are improving the understanding of cellular mechanisms and how biological systems respond to different perturbations. Currently, there is a need for novel computational methods that facilitate the analysis and integration of increasing volume of available data.

**Results:** In this paper, we present Totoro a new constraint-based approach that integrates quantitative non-targeted metabolomic data of two different metabolic states into genome-wide metabolic models and predicts reactions that were most likely active during the transient state. We applied Totoro to real data of three different growth experiments (pulses of glucose, pyruvate, succinate) from *Escherichia coli* and we were able to predict known active pathways and gather new insights on the different metabolisms related to each substrate. We used both the *E. coli* core and the iJO1366 models to demonstrate that our approach is applicable to both smaller and larger networks.

**Availability:** Totoro is an open source method (available at https://gitlab.inria.fr/erable/totoro) suitable for any organism with an available metabolic model. It is implemented in C++ and depends on IBM CPLEX which is freely available for academic purposes.

## 1 Introduction

The increasing availability of metabolomic data and their analysis are currently enhancing our knowledge on diverse biological mechanisms and elucidating how cells and organisms respond to different perturbations (Sevin et al., 2015). Metabolomics can be used to obtain a metabolic profile that characterizes the physiological response of a cell, tissue or organism to a stress or to a general perturbation (Roessner and Bowne, 2009), and experiments ranging from shorter-term responses (such as stress response programs) to longer-term responses (such as acclimation) are broadly available for diverse species. Different network-based strategies for metabolomic data analysis have been recently reviewed in (Perez de Souza et al., 2020) and amongst others, such strategies can be used to establish associations between metabolites or to integrate them into metabolic pathways.

Metabolic profiles are often analyzed and interpreted with the help of bioinformatic software such as MetExplore (Cottret et al., 2018; Frainay et al., 2019), MetaboAnalyst (Xia et al., 2015; Chong et al., 2018) or 3Omics (Kuo et al., 2013) that can identify the set of metabolites with a significant change in their concentration. The metabolomic data are projected on the annotated metabolic pathways in order to highlight the processes that may be linked to the observed changes. The aforementioned software also try to integrate different kinds of omic data (such as transcriptomic, metabolomic or proteomic data) in order to give a deeper understanding of the studied mechanisms (Cambiaghi et al., 2017). Different approaches were reviewed in (Rosato et al., 2018; Ivanisevic and Want, 2019; Stanstrup et al., 2019) and software for the enrichment analysis of metabolomic data were evaluated and their results compared in (Marco-Ramell et al., 2018). However, metabolic pathways have subjective definitions and can differ between databases (Ginsburg, 2009). Additionally, this kind of analysis can make it hard to identify the connections between metabolites since they can be part of many pathways and it is thus possible to miss paths which traverse several biological pathways.

Another approach is to use graph-based methods that allow to consider the whole metabolism as an integrated system focusing on the parts that are connecting the metabolites of interest. Usually, these methods rely mainly on the network structure, chemical information and on an input list of metabolites (Frainay and Jourdan, 2017). Another example can be seen in (Acuña et al., 2012; Milreu et al., 2014), with the enumeration of metabolic stories. A metabolic story is defined by the authors as the set of reactions that summarize the flow of matter from a set of source metabolites to a set of target metabolites and is characterized as a maximum directed acyclic subgraph connecting the metabolites of interest. One of the drawbacks of this approach is that a metabolic story is acyclic and thus, it is not possible to obtain sets of reactions that contain cycles. Nevertheless, cycles are common in metabolic networks and this assumption does not reflect reality. Additionally, the method does not take into account the stoichiometry of the reactions, which can lead to a set of unfeasible reactions in practice.

Metabolite concentrations have also been used to assess the responses to small perturbations in the context of constraint-based models (Palsson, 2000; Covert and Palsson, 2003; Klamt et al., 2014), and has been reviewed in detail by (Topfer et al., 2015). While standard flux balance analysis (FBA) tries to predict the flux distribution for one specific steady-state condition, dynamic FBA, as described in (Mahadevan et al., 2002), has been extensively used in smaller models to predict the evolution of the fluxes and of the metabolite concentrations over time. In (Reznik et al., 2013), the authors provide a method derived from the classical FBA framework, and showed that the variables of the dual problem (the so-called shadow prices, which correspond to the sensitivity of FBA to imbalances in the flux) can indicate if a metabolite is a growth-limiting metabolite in FBA. In (Bordbar et al., 2017) the authors describe the unsteady-FBA method (uFBA), created to integrate dynamic time-course metabolomics with a constraint-based metabolic model, allowing a bypass into the steady-state assumption for intracellular metabolites that are measured. In (Rohwer and Hofmeyr, 2008; Christensen et al., 2015), methods are presented to identify regulatory metabolites and paths by varying *in silico* their known concentrations in a measured steady-state using supply-demand analysis. Therefore, these methods are based on the response of an organism to a relatively small perturbation and on the influence of the metabolite concentrations on the reaction rates of the system to return to the original equilibrium.

In this paper, we focus not on the metabolite pools in one condition but on the difference of the obtained measurements between two conditions, which could be measured either within shorter or longer timeframes, depending on the biological question to be addressed. We also do not need neither comprehensive time-course datasets nor coupled data from the relative expression of genes or proteins, which are much harder to obtain. Our main hypothesis is that the difference of metabolite pools between two metabolic states can provide information on the transient state, that is, on the transition between the two measured conditions.

Similar problems have been studied in the literature. In (Sajitz-Hermstein et al., 2016), the authors provide a method (iReMet-Flux) to integrate relative metabolomic measurements in order to make predictions about differential fluxes. They use a constraint-based approach which minimizes the distance between the two flux vectors of the two different states based on the ratio between the measured metabolite concentrations in both conditions. For both states, steady-state is assumed for the flux vectors. However, the authors identify differential fluxes between the two conditions whereas we aim at finding reactions that are likely active during the transient state. In (Case et al., 2016), the authors investigated reachability problems in chemical reaction networks. Given two different states of the network, the goal is to identify a path that leads the network from the first state to the second one. They prove that this problem can be solved in polynomial time. However, they also discuss that a variant of this problem in which the maximum size of the path is fixed is more difficult to solve. Our approach overcomes this limitation at the same time that it minimizes the number of active reactions in the solutions, since we are interested in identifying only the parts of the network that are potentially active during the transient state. Even though other methods could be adapted to answer this problem, our objective is much simpler, requiring less computational complexity. By reformulating our problem in a simpler way we can also address larger genome-scale metabolic models, instead of focusing on smaller portions of the metabolism (e.g., core models).

We use constraint-based modeling to enumerate sets of reactions that explain the changes in concentrations for some measured metabolites, i.e., how the system moved from a state to another. We implemented our approach in a software we called Totoro (for “Transient respOnse to meTabOlic pertuRbation inferred at the whole netwOrk level”), that is publicly available at https://gitlab.inria.fr/erable/totoro, along with the test datasets presented in this study. It is implemented in C++ and depends on IBM CPLEX which is freely available for academic purposes. We also tested our method with data from pulse experiments with different carbon sources (glucose, pyruvate and succinate) in *Escherichia coli*.

## 2 Methods

A metabolic network can be represented as a weighted directed hypergraph H(V,R,S) where V is the set of vertices, R the set of hyperarcs and S the stoichiometric matrix representing weights on the hyperarcs. Each c∈V represents a metabolite of the network and each hyperarc r∈R a reaction that connects two sets of disjoint metabolites *Subs*_*r*_, *Prod*_*r*_ with $Subs_{r},Prod_{r}\subseteq V$ . To each hyperarc, a set of weights is associated representing the stoichiometric coefficients of the metabolites participating to the corresponding reaction. These weights are given by the stoichiometric matrix S which is a *m* × *n* matrix where each column represents a reaction and each row a different metabolite. It contains the stoichiometric coefficients which are positive if a metabolite is produced by a reaction and negative if it is consumed.

The set X⊆V contains all measured metabolites. The metabolomic data is given as a list which, for each measured metabolite in *X*, contains an interval. This interval describes by how much the internal metabolite concentration changed between two different states. Usually, small deviations for the measurements are available which can be used to calculate the minimum and the maximum possible difference between the internal metabolite concentrations. Furthermore, all reversible reactions of the network are split into forward and backward reactions.

**FIGURE 1:**
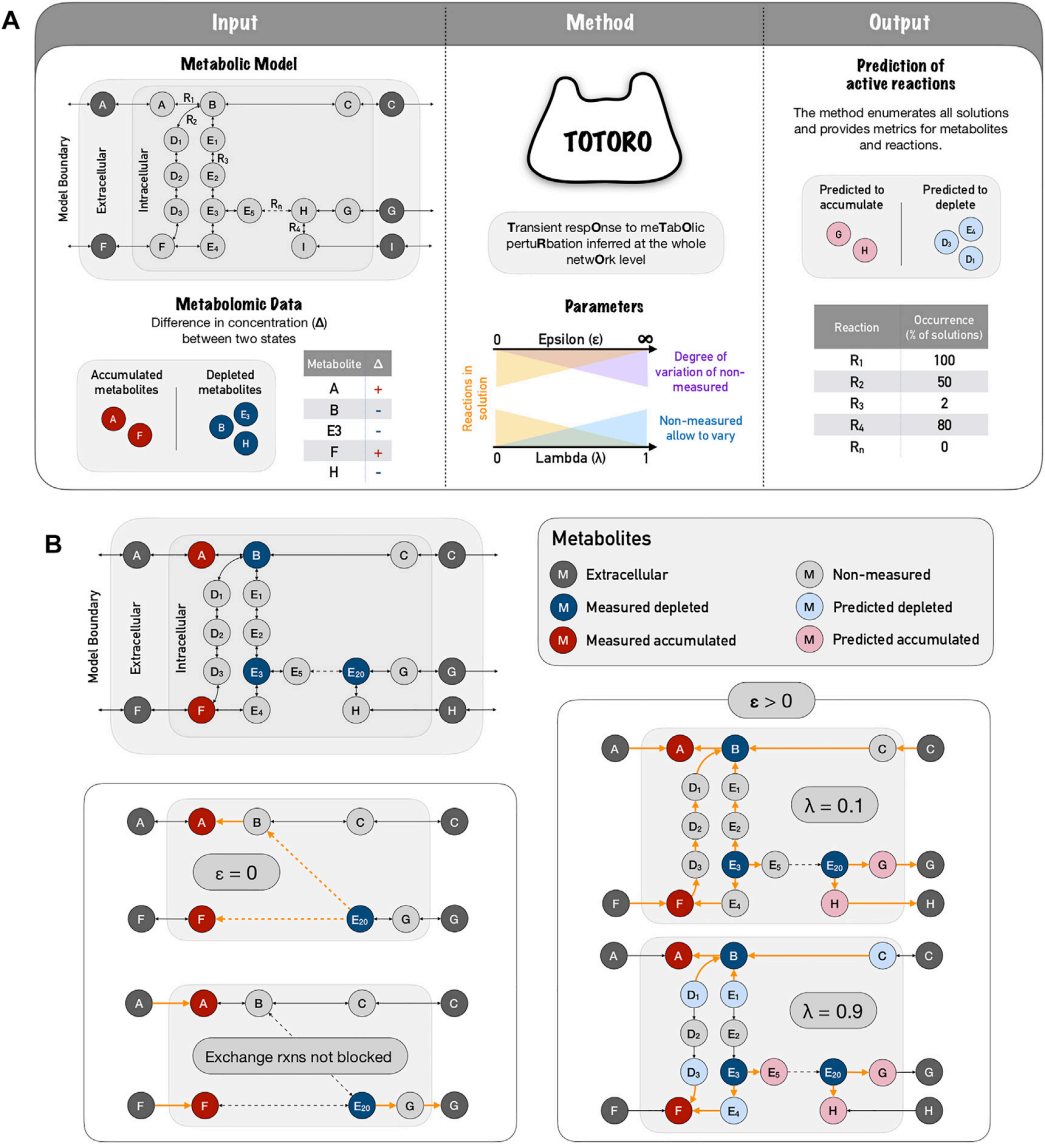
Totoro method explained. **(A)** Totoro is able to integrate a metabolic model with metabolomic data in order to predict active reactions during the transient state between two conditions (or simply after a perturbation). The inputs of Totoro are an SBML metabolic model, and a list of intervals for the difference in concentration (Δ) for each measured metabolite. In the metabolic model panel, grey circles depict metabolites and arrows depict reactions. In the metabolic data panel, accumulated metabolites are depicted in red circles, depleted metabolites are depicted in blue circles. The method Totoro then requires two additional user-defined parameters to fine tune the results, namely *λ* and *ϵ*. Totoro provides as output the predicted variation of metabolites and reactions that were most likely active between the two states in each enumerated solution as well as metric files grouping all enumerated solutions. In the figure, reaction occurrence is depicted as a percentage in all enumerated solutions. **(B)** The fine-tuning of parameters *λ* and *ϵ* are provided within a toy network, in which active reactions are showed in orange and dashed arrows indicate several reactions in a row. When we don’t allow an accumulation of non-measured metabolites (*ϵ* = 0), the method will try to connect the input deltas of distant and possibly unrelated metabolites; and in the case exchange reactions are not blocked, the method will most likely propagate the accumulation or depletion towards outside of the boundaries of the model. When accumulation is allowed (*ϵ* > 0) a low lambda (*λ* = 0.1) will favor solutions in which fewer non-measured metabolites accumulate or deplete, and will include a larger number of reactions within the solutions. As we raise the parameter lambda (*λ* = 0.9), we favor local and smaller solutions.

We are interested in solving the following problem: Given a network *H* and a list containing the changes for some metabolite concentrations before and after a perturbation, we want to identify sets of reactions that were involved in diverting the system from the initial state before the perturbation to the state after the perturbation (Figure 1A). Here, we present a constraint-based approach to solve this problem where the change of concentrations (Δ) between two states is represented as an interval.

### 2.1 Core Method

The variation of the concentrations in time of the metabolites in *X* can be written as:

$$\frac{dX}{dt}={\left(S\cdot v\right)}_{X}.$$ (1)

In this equation, *v* is a flux vector and the (⋅)_*X*_ operator means that only the entries of the vector corresponding to the metabolites in *X* are taken into account. We use [*X*]_*t*_ to denote the concentration for the metabolites in *X* at time point *t*. Considering two points *t*_0_ and *t*_1_ in time and $\Delta_{X}={\left[X\right]}_{t_{1}}-{\left[X\right]}_{t_{0}}$ , one can write:

$$\Delta_{X}=S\cdot \varphi .$$ (2)

In this case, each entry of the vector *φ* can be interpreted as the overall number of moles that passed through the reaction *j* during the time interval [*t*_0_, *t*_*f*_] which corresponds to the area under the reaction rate curve in this time interval:

$$\varphi_{j}=\int_{t_{0}}^{t_{1}}v_{j}\left(t\right)\cdot dt.$$ (3)

Due to biological and technical variability that can arise from different replicates of the same experiment, we assume that the measured variations in concentrations of the metabolites in *X* are represented by an interval rather than using a fixed number:

$$\Delta_{X}=\left[\Delta_{X}^{\min},\;\Delta_{X}^{\max}\right].$$ (4)

Furthermore, for the non-measured metabolites, we do not know if their concentration changed or not. Therefore, similarly to the approach of uFBA (Bordbar et al., 2017) and their ‘node relaxation’ to allow for changes in non-measured metabolites, we assume that a variation (*ϵ*) is possible for all non-measured metabolites $\bar{X}=V\backslash X$ :

$$\Delta_{\bar{X}}=\left[\epsilon^{\min},\;\epsilon^{\max}\right].$$ (5)

Based on these assumptions, we can model the production or consumption of metabolites between two states by the following constraints:

$$\begin{array}{ccc} & \Delta^{\min}\leq S\cdot \varphi \leq \Delta^{\max}\\ & 0\leq \varphi_{j}\leq u_{j} & \;\forall j\in R. \end{array}$$ (6)

All *φ*_*j*_ are positive and have an upper bound *u*_*j*_. We have that Δ^min^ is a vector composed of $\Delta_{X}^{\min}$ and *ϵ* ^min^ while Δ^max^ is composed of $\Delta_{X}^{\max}$ and *ϵ* ^max^.

As showed above, in our formulation, the variable *φ* can only be zero or have a positive value. For this, we use an additional constraint as explained in Section 2.2 in order to prevent both forward and reverse senses of reversible reactions from being picked in any given solution. However, this means that we do not know if the activity of the corresponding reaction was increased or decreased during the shift compared to the initial steady state. We only know that if *φ*_*j*_ is zero in the solution, reaction *j* is proposed as inactive during the shift while if *φ*_*j*_ has a non-zero value, reaction *j* is proposed as active during the shift. Hence, we are only interested in the reactions that have a non-zero *φ* because we want to identify the part of the metabolic network that was active during the metabolic shift. These reactions are represented by the support of the vector *φ*.

### 2.2 Minimizing the Number of Reactions and the Variation of the Concentrations for the Non-Measured Metabolites

Since the number of possible paths that can explain the measured metabolic shifts can be very large, we will focus on finding the smallest solutions with regard to the number of active reactions that still explain the metabolic shift. This corresponds to the parsimonious assumption that the fewest possible resources are used or the smallest changes are made. Thus, we are interested in identifying minimum sets of reactions that play a major role in the metabolic shift. For each reaction *j*, a binary variable *y*_*j*_ is then introduced that is set to zero if and only if the corresponding *φ*_*j*_ is zero and therefore, the reaction is not part of the solution. In this way, these variables will correspond to the support vector of *φ* and it will be sufficient to minimize their sum:

$$\begin{array}{ccc} & \;y_{j}=0↔\varphi_{j}=0\; & \forall j\in R\\ & \;y_{j}\in \left\{0,1\right\}. \end{array}$$ (7)

Additionally, to prevent that both a reaction *j* and its reversible $\bar{j}$ can be picked at the same time for one solution, the following constraint is used:

$$y_{j}+y_{\bar{j}}\leq 1\;\forall \left(j,\bar{j}\right)\in R.$$ (8)

To minimize the number of reactions that are part of the solution, the objective function is written as:

$$\min\overset{m}{\underset{j=1}{\sum }}y_{j}.$$ (9)

However, we are not only interested in minimizing the number of reactions in the solution but also in minimizing the variation in concentration for the non-measured metabolites $\bar{X}$ . Since the measured compounds are usually the more important ones for analyzing the biological experiment, it is reasonable to aim for solutions where other compounds do not accumulate or deplete a lot. This leads to the following minimization:

$$\min\underset{i\in \bar{X}}{\sum }|{\left(S\cdot \varphi \right)}_{i}|.$$ (10)

On the other hand, we are trying to explain as much change in the concentration as possible for the measured metabolites:

$$\max\underset{i\in X}{\sum }|{\left(S\cdot \varphi \right)}_{i}|.$$ (11)

To combine both ideas in one objective function, a weight *λ* is used for both objectives:

$$\min\lambda \overset{m}{\underset{j=1}{\sum }}y_{j}+\left(1-\lambda \right)\underset{i\in \bar{X}}{\sum }|{\left(S\cdot \varphi \right)}_{i}|-\left(1-\lambda \right)\underset{i\in X}{\sum }|{\left(S\cdot \varphi \right)}_{i}|.$$ (12)

The value for *λ* should lie between 0 and 1. Finding a good balance between these two objectives can be challenging but necessary to identify meaningful biological solutions (for a schematic representation of Totoro, see Figure 1A). A toy network example is provided in Figure 1B to show the influence of parameters *λ* and *ϵ* on the solutions. This will be further discussed in the following sections.

Summing up, the mixed-integer linear program (MILP) that is implemented in our software Totoro is the following:

$$\begin{array}{cc} \min_{\varphi ,y}\;\lambda \overset{m}{\underset{j=1}{\sum }}y_{j}+\left(1-\lambda \right)\underset{i\in \bar{X}}{\sum }|{\left(S\cdot \varphi \right)}_{i}|-\left(1-\lambda \right)\underset{i\in X}{\sum }|{\left(S\cdot \varphi \right)}_{i}| & \\ \begin{array}{ccc} s.t & \;\Delta^{\min}\leq S\cdot \varphi \leq \Delta^{\max}\\ & \;0\leq \varphi_{j}\leq u_{j} & \forall j\in R\\ & \;y_{j}=0↔\varphi_{j}=0 & \forall j\in R\\ & \;y_{j}+y_{\bar{j}}\leq 1 & \forall \left(j,\bar{j}\right)\in R\\ & \;y_{j}\in \left\{0,1\right\};\lambda \in \left(0,1\right);u_{j},\varphi_{j}\in R. \end{array} \end{array}$$ (13)

### 2.3 Enumerating Different Solutions

To enumerate different solutions, once a solution is found, it must be excluded for the next iteration. Two solutions are different if they do not contain the same reactions. We are using the following constraint where y* is a previously found solution vector:

$$\underset{j\in R:y_{j}^{*}=1}{\sum }y_{j}\leq \overset{m}{\underset{j=1}{\sum }}y_{j}^{*}-1.$$ (14)

This prevents that the exact same combination of reactions gets chosen again. Afterwards, we can solve the updated MILP again to compute a different solution. We repeat this process until no more new solutions can be found or until a desired number of solutions has been computed.

### 2.4 Dealing With Source/Sink Reactions and Non-Measured Metabolites

Source and sink reaction (i.e., reactions that have only products or only substrates) of the network should be blocked to avoid that changes in the concentration are just transferred outside of the network where they cannot be taken into account by the objective function. However, no information is lost if source and sink reactions are blocked. If the substrates of a sink reaction are accumulated or the products of a source reaction are depleted in a solution, this indicates that the corresponding source/sink reaction is active. Their use is limited by the chosen *ϵ* but it can be set to a very low or large value to imitate an infinite source or sink. Hence, specific sources or sinks can be added to the problem by specifying a large negative Δ^min^ or a large positive Δ^max^ for certain metabolites, but the method will remain robust to small variations, as long as the range of this parameter remains within a similar order of magnitude of the values of the measured metabolites.

However, if the minimization of the number of active reactions is prioritized (*λ* ≈ 1) and the value of *ϵ* for the non-measured metabolites is higher than the one for the measured metabolites, the changes in concentration of the measured metabolites can simply be distributed to (accumulated on or taken from) the nearby non-measured metabolites (Figure 1B, *ϵ* > 0, *λ* = 0.9) and prevents that larger sub-hypergraphs are chosen (which would instead connect several measured metabolites and explain how the depletion of one measured metabolite leads to the accumulation of another measured metabolite, or vice-versa). However, this can be addressed by decreasing the value of *λ* in the objective function and thereby giving more weight to the portion of the function that minimizes the accumulation in non-measured metabolites Figure 1B, *ϵ* > 0, *λ* = 0.1). This should result in solutions that are larger but that connect the measured metabolites better than when only the number of reactions is minimized. Furthermore, based on other experimental data, the user might choose smaller values of *ϵ*, or constrain it to the highest measured metabolite to further restrict the accumulation/depletion of the non-measured metabolites.

## 3 Results

To evaluate our approach, we used data from different pulse experiments with different carbon sources in *E. coli* as presented in (Taymaz-Nikerel et al., 2013). The authors measured the internal concentrations for several metabolites for a glucose baseline and for glucose, pyruvate and succinate pulse experiments. These data were used to apply the method on the *E. coli* core model (Orth et al., 2010) and the *E. coli* iJO1366 model (Orth et al., 2011) available from the BiGG database (King et al., 2015b). The *E. coli* core model consists of 72 metabolites and 95 reactions, the *E. coli* iJO1366 model of 1,805 metabolites and 2,583 reactions.

We were interested in the difference between the glucose baseline and the pseudo-steady state which was achieved in about 30–40s after each pulse. In (Taymaz-Nikerel et al., 2013), the authors provided the internal concentrations for the baseline, including the deviations for their measurements and the fold changes for the three different pseudo-steady states which we used to calculate the internal concentrations for each pseudo-steady state. In (Taymaz-Nikerel et al., 2013), deviations for the measured concentration of the glucose baseline are given that were derived from several replicates of the same experiment. We used them to be able to calculate the minimum difference $\Delta_{X}^{\min}$ and maximum difference $\Delta_{X}^{\max}$ in the concentrations between the glucose baseline and each pseudo-steady state. A detailed explanation can be found in the Supplementary Material Section S1.1. The calculated $\Delta_{X}^{\min}$ and $\Delta_{X}^{\max}$ for all three pulse experiments can be found in the Supplementary Tables S1–S3.

We used all measured metabolites that are present in the network and that had a significant change in their concentration as input. It should be noted that a change for each given metabolite must be either positive or negative. For further details, see the Supplementary Material Section S1.1.

Furthermore, source and sink reactions cannot be chosen as part of the solution and therefore glucose, pyruvate and succinate were added as sources for the corresponding pulse experiments. Oxygen was added as another source because in (Taymaz-Nikerel et al., 2013), the authors identified increased oxygen uptake rates during the pulse experiment. To allow unlimited growth, the biomass was added as sink.

**FIGURE 2:**
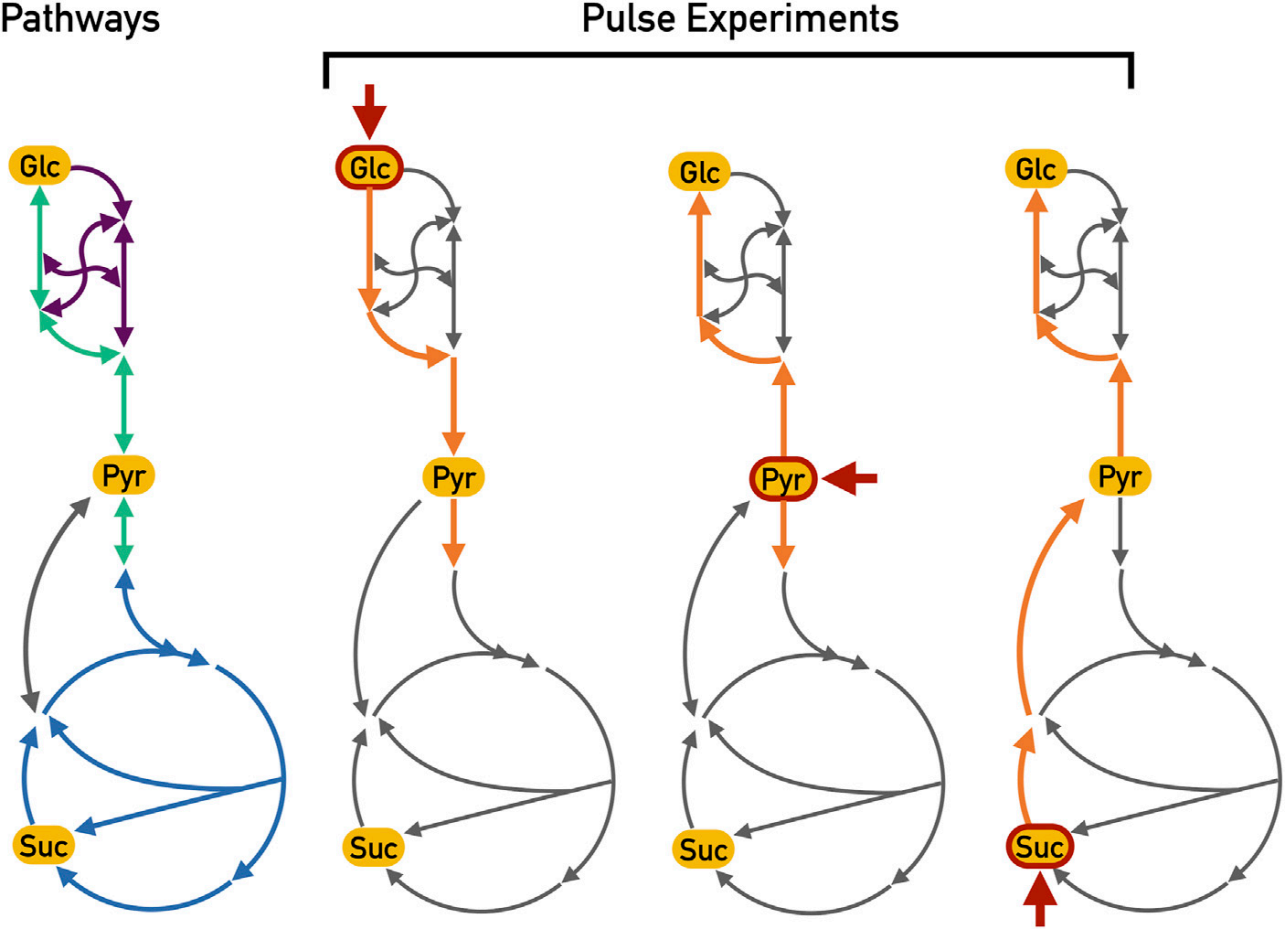
Expected active reactions for different pulse experiments. These essential reactions along with their expected directions are highlighted in orange whereas other non-essential reactions (but which nonetheless could be chosen) are depicted in grey. Each pulse is indicated by the short red arrow (Glc: glucose; Pyr: pyruvate and Suc: Succinate). During the glucose pulse, the glycolysis reactions (depicted in green) should be active in order to generate ATP from the hydrolysis of glucose. On the other hand, the pyruvate and succinate pulse experiments should show gluconeogenesis activation (also depicted in green but in the opposite sense), generating glucose-6-phosphate from these two carbon sources. Furthermore, the TCA cycle (depicted in blue) can be fed from pyruvate during the pyruvate and glucose pulses. During the succinate pulse, the overflow in the TCA cycle should lead to the production of pyruvate with a subsequent activation of gluconeogenesis to produce biomass precursors. The pentose phosphate pathway (depicted in purple) is most likely active in all pulses in order to generate biomass precursors; however, since this pathway is a mere interconversion of carbohydrates, there is no particular expectation as to the actual direction of these reactions.

The expected active reactions in the core metabolism of *E. coli* are displayed in Figure 2 for each pulse experiment.

### 3.1 *E. coli* Core Model

At first, the method was applied using the *E. coli* core model. To better understand how the different parts of our model impacted the solutions, we did several runs with different values for *λ* (0.1, 0.5, and 0.9) and *ϵ* (5 and 10) for each pulse experiment. Although a single solution should be enough to identify some pathways responsible for the shift, we wanted to see if we could also obtain alternative pathways. Furthermore, we wanted to investigate how the solutions evolve when they are slightly suboptimal. For each different parameter setting, 100 different solutions were therefore enumerated. The results are displayed using *Escher* (King et al., 2015a) in the Supplementary Figures S1to S18.

In general, we could observe that solutions with *λ* = 0.1 were preferable since usually the goal is to have a final solution which is overall more connected. In this way, we were able to extract connected sub-hypergraphs that resemble complete biological pathways which played a role during the metabolic shifts. This was the case for all three pulse experiments. A higher *λ* led to solutions that were less connected since the optimizer prioritizes solutions with fewer active reactions, and depending on the case, it might be harder to interpret these solutions biologically. Nevertheless, the user is able to fine-tune the number of reactions in the final solution and the degree of connectivity (for instance, if the goal is to highlight only parts of the complete metabolic network instead of finding a connected sub-hypergraphs).

By adjusting the parameters *λ* and *ϵ*, Totoro could propose connected sub-hypergraphs for all three pulse experiments. The predicted solutions did not use co-factors as shortcuts through the network. We therefore did not modify our method further to treat co-factors separately.

#### 3.1.1 Pyruvate Pulse

**FIGURE 3:**
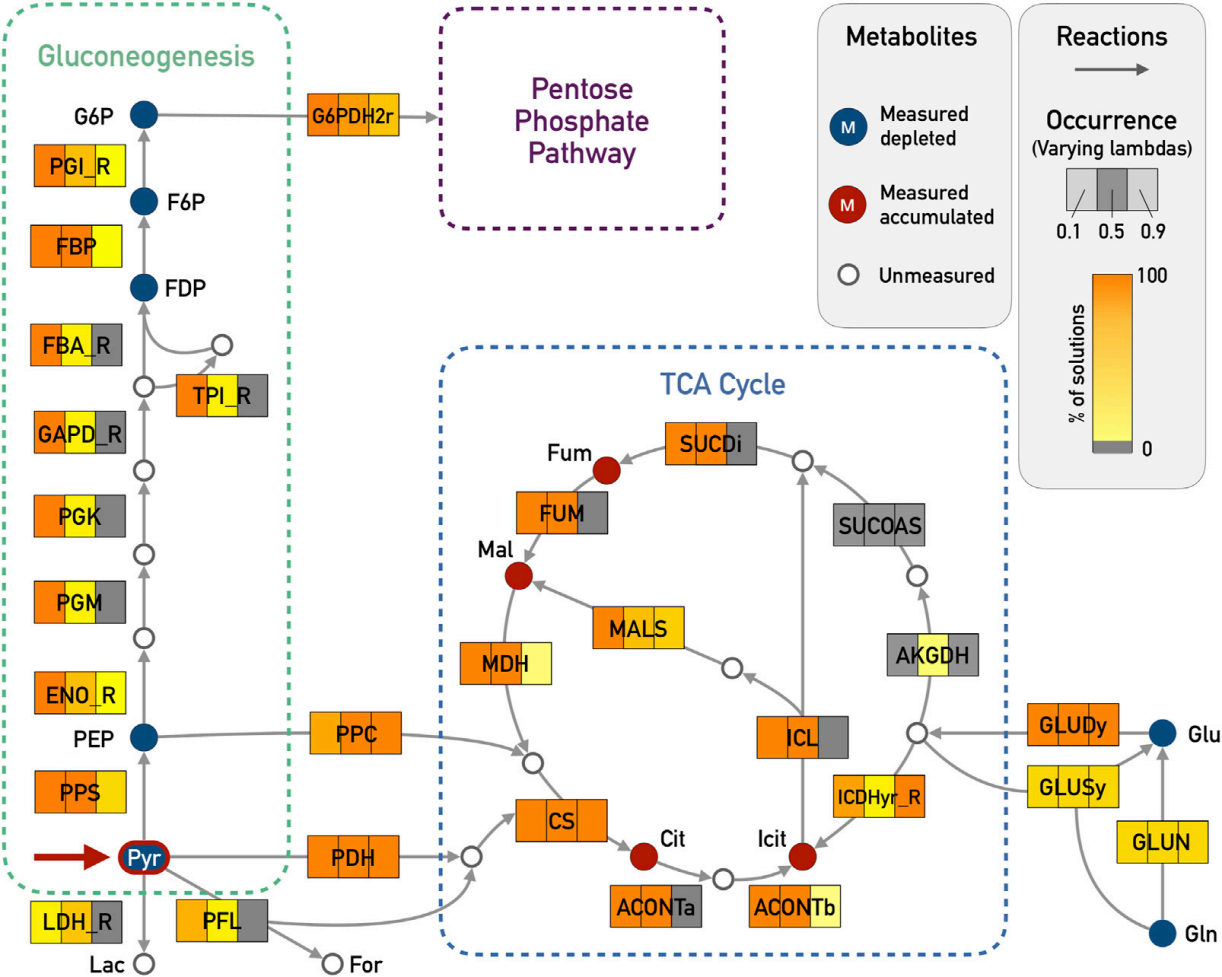
*E. coli* core model - Results for Gluconeogenesis and TCA Cycle in the pyruvate pulse (red arrow in Pyr) with *ϵ* = 5 and varying *λ* (0.1, 0.5, and 0.9). The metabolites that were given as input are highlighted in blue if the corresponding input deltas were below zero and red if they were above zero. Reactions that are highlighted in orange were chosen in almost all of the enumerated solutions, while light yellow corresponds to very few occurrences (less than 5%). Reactions in gray were not chosen in any solution. The expected reactions of the gluconeogenesis and part of the TCA cycle are active in all 100 solutions for *λ* = 0.1. The reversible reactions of the gluconeogenesis were chosen in the correct direction. For simplicity reasons, side compounds and cofactors were excluded from the figure. Abbreviations for metabolites: G6P, glucose-6-phosphate; F6P, fructose-6-phosphate; FDP, fructose-biphosphate; PEP, phosphoenolpyruvate; Pyr, pyruvate; Lac, lactate; For, formate; Mal, malate; Fum, fumarate; Cit, citrate; Icit, isocitrate; Glu, glutamate; Gln, glutamine; Abbreviations for reaction names (_R indicates the reverse direction of a reversible reaction within the original model): G6PDH2r, glucose 6-phosphate dehydrogenase; PGI, glucose-6-phosphate isomerase; FBP, fructose-bisphosphatase; FBA_R, fructose-bisphosphate aldolase; TPI, triose-phosphate isomerase; GAPD, glyceraldehyde-3-phosphate dehydrogenase; PGK, phosphoglycerate kinase; PGM, phosphoglycerate mutase; ENO, enolase; PPS, phosphoenolpyruvate synthase; LDH, D-lactate dehydrogenase; PFL, pyruvate formate lyase; PPC, phosphoenolpyruvate carboxylase; PDH, pyruvate dehydrogenase; CS, citrate synthase; ACONTa, Aconitase (half reaction A); ACONTb, Aconitase (half reaction B); ICDHyr, Isocitrate dehydrogenase; AKGDH, 2-Oxoglutarate dehydrogenase; SUCOAS, Succinyl-CoA synthetase; SUCDi, Succinate dehydrogenase; FUM, fumarase; MDH, malate dehydrogenase; ICL, isocitrate lyase; MALS, malate synthase; GLUDy, glutamate dehydrogenase; GLUSy, glutamate synthase; GLUN, glutaminase.

For the pyruvate pulse, we expected that the activity of the TCA cycle would increase and that reactions for gluconeogenesis would be active (see Figure 2). Both observations could be reproduced with a *λ* = 0.1 (see Figure 3 for a comparison of the values of *λ*), while higher values of lambda constrained the solutions locally around the measured metabolites. For *λ* = 0.9, neither the TCA cycle nor the gluconeogenesis pathway were proposed to be active. Setting *λ* to 0.5 already improved the results: the TCA cycle was proposed as active but the complete gluconeogenesis pathway was only recovered in less than 50% of the solutions.

The four measured metabolites citrate, isocitrate, L-malate and fumarate had positive input deltas and could thus be used as sinks. The results showed how the TCA cycle can be fed from pyruvate either by the phosphoenolpyruvate carboxylase (PPC) or by the combination of pyruvate dehydrogenase (PDH) and citrate synthase (CS). Furthermore, the pathway from pyruvate to glucose 6-phosphate (G6P) was active in 100% of solutions for *λ* = 0.1. The pathway from pyruvate to G6P contains nine reactions including seven reversible ones: glucose-6-phosphate isomerase (PGI), fructose-bisphosphate aldolase (FBA_R), triose isomerase (TPI), glyceraldehyde-3-phosphate dehydrogenase (GAPD), phosphoglycerate kinase (PGK), phosphoglycerate mutase (PGM) and enolase (ENO). Especially here, it is important to state that all these reversible reactions were predicted in the correct direction going from pyruvate towards G6P. The core network results can be seen in Supplementary Figures S1–S6, with varying *λ* and *ϵ*. These figures were created using *Escher* (King et al., 2015a).

We do not fix the objective value in our optimization problem after obtaining the first solution but in every iteration, the minimization problem is solved again after excluding the newly found solution. This means that the next solution can have the same objective value but it is also possible that the objective value is worse than in the previous iteration. In this particular case, the 100th solution had an objective value that was only 5.5% worse than the objective value of the first solution (see Table 1) which shows that, as concerns optimality, all 100 solutions were very similar. They also had very similar active reactions. Comparing the 100 enumerated solutions for *λ* = 0.1 and *ϵ* = 5, a total of 43 reactions with a specific direction were chosen in all solutions. Out of these 43 reactions, 24 were chosen in every solution (including reactions in the TCA cycle and the gluconeogenesis pathway). This means that certain core pathways were consistently picked also in slightly suboptimal solutions. Looking at only the ten best solutions, already 38 out of the 43 reactions were identified. The missing reactions were mostly part of the pentose phosphate pathway which also contains reactions that were part of the solution only in a few cases. Even with only ten solutions, we were able to obtain the alternative pathways feeding the TCA cycle (PPC/PDH). This indicates that it is not necessary to enumerate a large amount of solutions to get significant results and to identify alternative biological pathways.

**TABLE 1:** Comparison of different objective values for the best runs for each experiment. Since we are not fixing the objective value of the first solution in our optimization problem, the objective values for the subsequent solutions can be worse. In this table, we are comparing the difference in the objective values between the first solution and the 100th solution. In addition to the absolute differences, also the percentage of how much the objective value worsened compared to the first solution is displayed. The underlying optimization problem is a minimization problem. Therefore, smaller objective values are better.

Pulse experiment	1st sol.	100th sol.	Abs. diff.	%
Pyruvate (*λ* = 0.1, *ϵ* = 5)	−32.139 4	−30.663 5	1.48	5.5
Glucose (*λ* = 0.1, *ϵ* = 1.2)	5.383 0	6.558 2	1.18	21.8
Succinate (*λ* = 0.1, *ϵ* = 5)	−158.177 0	−157.576 0	0.60	0.4

To check the robustness of the method against small perturbations, we tested within the pyruvate pulse the results of Totoro for the values of *λ* = 0.1 and 0.9, excluding one metabolite at a time, recomputing the results, and computing the distances to the results on the complete metabolite set for reaction occurrence (in terms of absolute difference of occurrences). Overall, the results for both *λ* = 0.1 and 0.9 differed from less than 5% to around 20%. In general, the results were robust ( <10% in average distance) for 70–80% of the metabolites tested (with *λ* = 0.1, and 0.9, respectively), but we noticed that excluding metabolites with a higher neighborhood connectivity (such as glutamate and glutamine) had a greater impact on the final results. These results show that even though the distances were small, the amount of information provided by different metabolites varied widely.

Moreover, we tested 10 random sets of measured metabolites (Supplementary Tables S4, S5), with a varying number of excluded metabolites to detect at which point the method would not behave as with the complete dataset. In accordance with the previous results for single exclusions, and within the tests with less than 50% of the measured metabolites excluded, the smallest distance (≈10%) to the complete results came from a random dataset which included glutamate, glutamine and pyruvate (to ensure the carbon source uptake). As expected, when more than 50% of the measured metabolites were excluded, we detected a much higher difference (≈40%) between the results from the complete dataset and those from the random datasets.

#### 3.1.2 Glucose Pulse

For the glucose pulse, we expected that reactions that are part of the glycolysis pathway would be active as they convert glucose into pyruvate generating energy. Consequently, the TCA cycle should also be fed (see Figure 2). For *λ* = 0.9 and 0.5, the active reactions proposed by Totoro were disconnected and it was not possible to identify active pathways. We believe that the results coming from this pulse were less insightful since the bacteria were already grown in glucose prior to the pulse, which in turn might be a reason why the changes in metabolites were not as informative as the other pulses. This was for the most part corrected if more metabolites were added as input to Totoro when using the complete network as presented in Section 3.2. This also shows the importance of careful experimental design and how subtle perturbations may generate results that are not always homogeneous.

Even for *λ* = 0.1 and *ϵ* = 5, only disconnected parts of the network were active (see Supplementary Figure S9). Since we were interested in testing the method to obtain more connected sub-hypergraphs, we decided to fine-tune the solutions by lowering the value of *ϵ* as much as possible. The result for *ϵ* = 2 and 1.2 can be found in Supplementary Figures S10, S11, respectively. Lowering the value of *ϵ* to 1.1 rendered the underlying optimization problem infeasible. For *ϵ* = 1.2, we got solutions that linked intermediate metabolites of the glycolysis pathway to the TCA cycle through the PPC reaction. In some solutions, the TCA cycle was also fed by PDH and CS to account for the accumulation of citrate. As previously mentioned, when the solutions are disconnected and this is unwanted, decreasing the value of *ϵ* can sometimes help to obtain more connected solutions. However, this should be used carefully in order to avoid linking unrelated and distant metabolites, which might not be meaningful biologically.

The 100 solutions were very similar (*λ* = 0.1, *ϵ* = 1.2). They accounted for a total of 47 reactions (with distinct directions) and 30 of these appeared in all solutions. Similarly to the pyruvate pulse, the difference in these solutions were mostly based on a few reactions that are not part of the main pathways (glycolysis/TCA cycle). One critical observation is that the D-glucose transport reaction (GLCpts) was not part of every solution although glucose should be used as important source. As previously mentioned, the bacteria were already grown in glucose prior to the glucose pulse, which is possibly a reason why glucose was already internalized prior to the initial pulse. When comparing the objective values for these 100 solutions, the absolute difference between the first solution and the 100th one was similar to the one observed for the pyruvate pulse (see Table 1). However, proportionally this value was 21.8% worse than for the first solution. When we repeated the run for *λ* = 0.1 and *ϵ* = 1.2 with 50 iterations, the D-glucose transport reaction was part of 42 solutions. For ten iterations, this reaction was picked in all ten solutions. Hence, the glucose transport reaction was active in solutions with the best objective values. This showed that although the solutions remained very similar, there was a decline in their quality. And similarly to the pyruvate pulse, we saw that it is not necessary to enumerate a large amount of solutions.

#### 3.1.3 Succinate Pulse

After the succinate pulse, part of the TCA cycle should always be active. Furthermore, the gluconeogenesis pathway should be active to produce G3P and glucose-6-phosphate from succinate. Again, the results for *λ* = 0.5 and 0.9 led to smaller solutions that were more disconnected (see Supplementary Figures S13–S16). Therefore, we focused on the analysis of the results for *λ* = 0.1 (see Supplementary Figures S17, S18). For both *ϵ* = 5 and 10, succinate entered the TCA cycle and turned into oxaloacetate. Totoro proposed two possibilities to output the excess of the TCA cycle: Either phosphoenolpyruvate (PEP) was produced by PEP carboxykinase (PPCK) or by PEP synthase (PPS) using pyruvate as intermediate substrate. Subsequently, PEP was, as expected, transformed to G3P. The lower right part of the TCA cycle predicted as active can be explained by the fact that the concentration of L-glutamate decreased and the concentration of citrate increased. The active reaction in this part connected these two metabolites. Furthermore, reactions of the pentose phosphate pathway were proposed as active and the biomass precursors R5P, E4P, and G3P were produced.

The results for *ϵ* = 5 and 10 were very similar. For example, one difference was that for *ϵ* = 10, the reverse D-lactate dehydrogenase (LDH) was predicted to be active in 56 solutions which led to a small accumulation of D-lactate. It does make sense biologically because in general, D-lactate is one of the main products of the fermentation but we do not have the measurements for the concentration of D-lactate for this pulse experiment to actually verify this observation. However, in total, the differences were negligible and in contrast to the glucose pulse, the parameter *ϵ* had a lower impact on the outcome.

Again, the core reactions of all 100 solutions were very similar. In total, 41 reactions (with distinct directions) appeared in all 100 solutions (for *λ* = 0.1, *ϵ* = 5). We observed that 22 of these were always active (mostly in the gluconeogenesis pathway and part of the TCA cycle). The objective values for all 100 solutions were extremely close (see Table 1).

### 3.2 *E. coli* iJO1366 Model

Based on the results for the *E. coli* core model, we only did runs with *λ* = 0.1 for the *E. coli* iJO1366 model. The inputs were updated because this network contains more metabolites and therefore, more measured metabolites could be added. The amount of iterations was decreased to ten because the runtime in the larger network is significantly higher and we had already established in the core model that it was not necessary to enumerate a larger amount of solutions. To decrease the runtime for each solution, CPLEX was configured differently. The relative MIP gap tolerance was set to 0.05 which means that the solver will stop an iteration if a solution is found that is within 5% of the optimal. This allows for a faster result and we could see in the core model that the first 100 solutions tended to be very similar. This means that even if we are enumerating slightly suboptimal solutions, we should be able to compute solutions that are very similar to the actual optimal solution. If the 5% limit is not reached after 48 h, the iteration is stopped. The memory usage of CPLEX was limited to 10 GB.

The runtime for the different pulse experiments differed a lot. The results for the pyruvate and glucose pulses were computed on a cluster. For the pyruvate pulse, the 5% limit was reached only in three iterations (see Supplementary Table S6). All other iterations were stopped after 48 h. However, all solutions obtained were within 7% of the optimum. Thus, we still took them into account when analyzing the predicted active reactions. In none of the iterations for the glucose pulse, the 5% limit was reached. The obtained solutions were within 8.5% of the optimal value (see Supplementary Table S7).

In contrast to the pyruvate and the glucose pulses, the 5% limit was reached in all iterations for the succinate pulse and computing all ten solutions took less than 5 min on a personal machine (2.90 GHz Intel i7-7820HQ CPU, 16 GB RAM). This shows that the constraints describing the input deltas in the MILP have a large influence on the difficulty of the optimization problem, and thus also on the runtime.

However, although the obtained solutions were suboptimal, the active reactions predicted by Totoro for the core metabolism were similar to the best results of the *E. coli* core model for all three pulse experiments. For instance, in the pyruvate pulse results, out of 12 reactions in the TCA cycle within the large network, 8 were also present in the core model. In total, 5 were chosen in 100% of the solutions in the same direction in both core and large networks. The complete network was also able to correct the only inconsistency within the TCA cycle for the core network: the direction of the reaction ICDHyr, which shows the advantage of relying on complete networks whenever available. For the glycolysis/gluconeogenesis pathways, out of 12 reactions, 9 were also included in the core model. In total, 6 reactions were chosen in 100% and 1 in more than 80% of the solutions in the same direction in both networks. Totoro predicted as active for pyruvate, glucose and succinate (in at least 1 solution) a total of 221, 284, and 189 reactions respectively. Moreover, 52% of the reactions were chosen across all iterations in the pyruvate pulse dataset, 81% in the succinate pulse dataset and 62% in the glucose pulse dataset.

**FIGURE 4:**
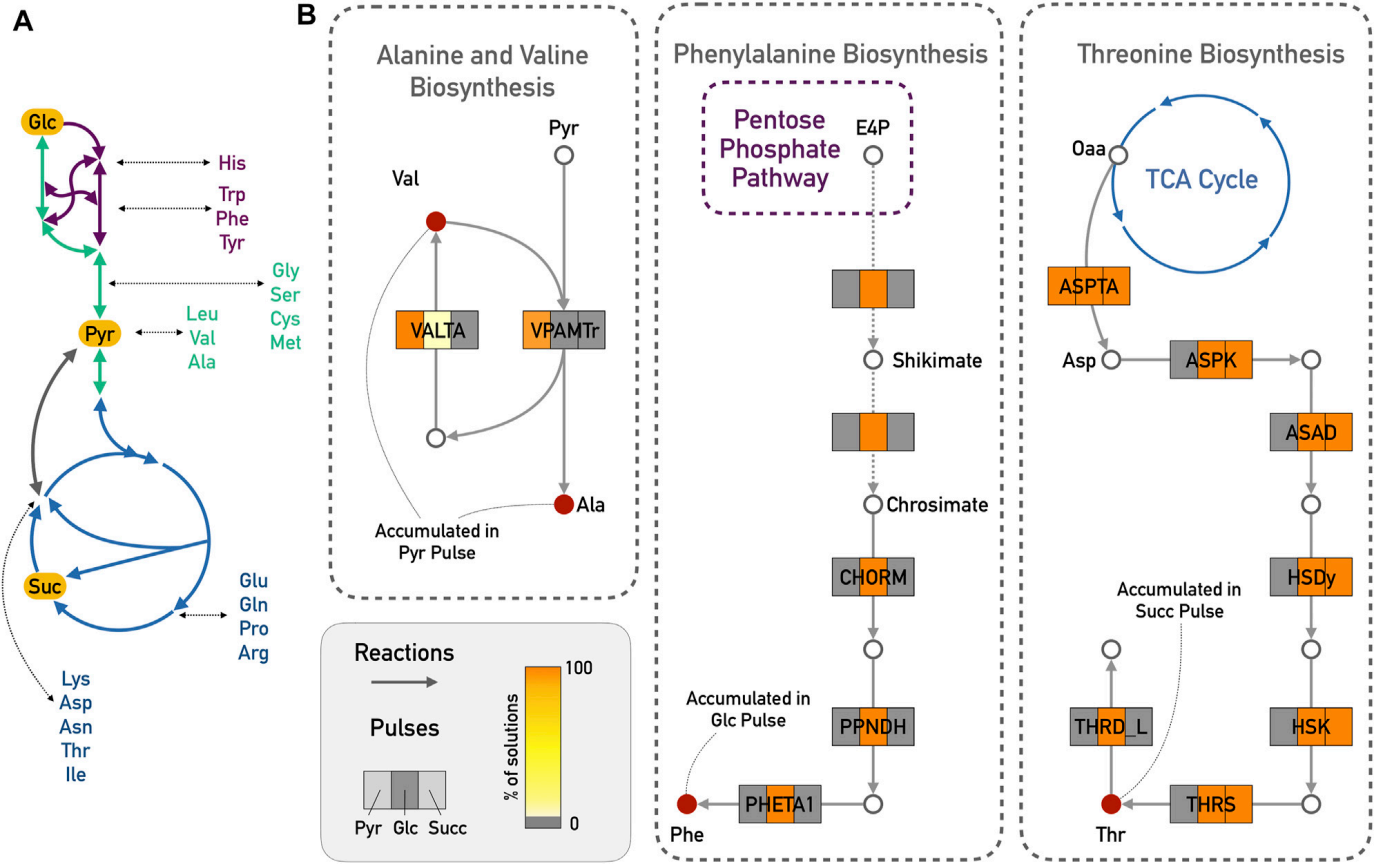
Amino acid biosynthesis in the *E. coli* iJO1366 model. **(A)** Schematic representation of carbon sources with closely related amino acids. Glycolysis/Gluconeogenesis in green; TCA cycle in blue and Pentose Phosphate Pathway in purple. **(B)** Totoro results explaining the accumulation of valine (Val) and alanine (Ala) in the pyruvate (Pyr) pulse; accumulation of phenylalanine (Phe) from the glucose (Glc) pulse and accumulation of threonine (Thr) from the succinate (Succ) pulse. For simplicity reasons, side compounds and cofactors were excluded from the figure. Dashed arrows indicate several reactions from the shikimate and chorismate pathways. Abbreviations for reaction names are as follows: VALTA, valine transaminase; VPAMTr, valine-pyruvate aminotransferase; CHORM, chorismate mutase; PPNDH, prephenate dehydratase; PHETA1, phenylalanine transaminase; ASPTA, aspartate transaminase; ASPK, aspartate kinase; ASAD, aspartate-semialdehyde dehydrogenase; HSDy, homoserine dehydrogenase; HSK, homoserine kinase; THRS, threonine synthase; THRD_L, L-threonine deaminase.

The additional measurements that were added as input deltas for the large network were mostly amino acids (see Supplementary Tables S1–S3). In (Waschina et al., 2016), the authors show for the example of amino acid production in *E. coli* how the production cost for individual amino acids can depend on the available carbon source, and reactions close to the entry point of the carbon source might have considerably higher fluxes. A schematic representation of this is provided in Figure 4A. Indeed, from the experimental data, alanine and valine only accumulated during the pyruvate pulse, and were depleted with the other two carbon sources. Pyruvate is a direct precursor for valine production. We therefore expected that reactions of the alanine and valine biosynthesis should play a greater role in the predicted results for pyruvate compared to the other two pulses. Totoro predicted an activation of the pathway from pyruvate to alanine and valine, which resulted in the accumulation of these amino acids (Figure 4B). In accordance with the predictions in (Waschina et al., 2016), another example is the accumulation of threonine during the succinate pulse. Threonine and succinate are closely connected, and Totoro predicted active reactions leading to its biosynthesis and accumulation in the succinate pulse (Figure 4B). Compared to the results for succinate, Totoro predicted more active reactions consuming threonine during the glucose pulse, and no reactions producing it in the pyruvate pulse, resulting in the depletion of this amino acid with those carbon sources. Moreover, only during the glucose pulse, phenylalanine was accumulated, and Totoro proposed the complete pathway for the phenylalanine biosynthesis as active when compared to the pyruvate and succinate pulses (Figure 4B), in accordance with the predictions in (Waschina et al., 2016) of lower cost to produce this amino acid with glucose as carbon source.

## 4 Discussion

Totoro was able to predict expected pathways as active based on the differences in the measured concentrations for some internal metabolites for both the *E. coli* core and complete models. We show that in general, it is preferable to use smaller values of *λ* (e.g., *λ* = 0.1) though the method is not critically sensible to this setup, being robust to small perturbations. However, it is worth noting that a higher *λ* can lead to smaller solutions which might be biologically irrelevant. Here, we focused in extracting connected sub-hypergraphs that explained the changes in concentration between two different conditions. We also show that a reduction of *ϵ* can also be used to obtain more connected solutions. However, there might be situations where the user might be interested in only local changes around the measurements. In this context, it might be advantageous to choose higher values for *λ* and *ϵ*. We did not encounter problems specific to co-factors which is a known problem when looking for shortest paths in metabolic networks. This is probably due to the fact that we are not only minimizing the number of active reactions in the solutions but also focusing on the changes in the metabolite concentrations. By splitting reversible reactions, Totoro was able to predict distinct directions for them.

Both in the core network and in the larger network, we were able to recover biologically meaningful pathways. Additionally, although the larger network contains more reactions and we added more input deltas, the predictions for the core metabolism of *E. coli* were fairly similar to the results for the core network. We also showed a particular case in which the perturbation was subtle, and the results from the complete model were more insightful than the ones from the core model. It must be however noted that the predictions do depend on the measured metabolites. If for large parts of the network, no metabolite concentrations are measured, Totoro will likely not be able to find active pathways for these parts of the network.

Moreover, we could also see that it is not necessary to enumerate a high number of solutions which is especially important when larger networks are used and the runtime of Tororo increases. We enumerated 100 different solutions for the core network. However, in our case, the enumerated solutions were very similar and a large amount of reactions appeared in all 100 solutions. Therefore, already one (or few) solution(s) would have been sufficient to infer the most important reactions that were proposed to be active.

## 5 Conclusion

In this paper, we presented Totoro, a method that identifies active reactions during the transient state based on the differences in the concentrations for some measured metabolites from two different conditions and we showed its prediction power on the example of different pulse experiments in *E. coli*. It is important to note that even though we provided several biologically trivial results, Totoro only used metabolomic data as basis for these predictions, without any other source of bias such as defined metabolic pathways. Our method was also able to handle full networks which take into account model stoichiometry, and we did not perform any type of filtering for cycles, reversible reactions or co-factors.

With the current technologies, it gets more common to have different kinds of data available which creates a need for methods that combine, for instance, metabolomic, transcriptomic and proteomic data. We have recently developed a method for integration of metabolic networks and transcriptomic data (Pusa et al., 2019) and we intend in the future to adapt our approaches to be able to integrate multiple kinds of omic data, similarly to what was proposed in (Pandey et al., 2019) for thermodynamic, transcriptomic and metabolomic data, and in (Kleessen et al., 2015) for transcriptomic and metabolomic data. On a larger scale, it might be interesting also to consider whether some measures used in (hyper)graph theory such as connectivity or (hyper)path length might be related to the parameters used and thus provide an automatic and perhaps more reliable way of setting them. Notice that achieving this would be even more challenging in the case of hypergraphs for which such measures might have to be adapted.

## Data Availability

The original contributions presented in the study are included in the article/Supplementary Material, further inquiries can be directed to the corresponding author.
