# Supplementary material for: Totoro: Identifying Active Reactions During the Transient State for Metabolic Perturbations
Source: Front Genet. 2022 Feb 21;13:815476. doi: 10.3389/fgene.2022.815476 (PMC8905348; doi:10.3389/fgene.2022.815476)
Supplement: Supplementary file 1 [file DataSheet1.pdf]

Supplementary Material

”Totoro: Identifying active reactions during the transient state for metabolic perturbations” by Ferrarini, Ziska and Andrade *et al.*, 2021.

1 SUPPLEMENTARY METHODS

1.1 Input deltas

The input deltas were calculated in the following way: For each measured metabolite X, we computed the minimum (maximum) internal concentration for the pseudo-steady state $[X]_{pss-min}$ ($[X]_{pss-max}$) using the baseline glucose concentration $[X]_{baseline}$ with the given deviations d_X and the fold changes f_X:

$$
\begin{aligned}
[X]_{pss-min} &= ([X]_{baseline} - d_X) \cdot f_X \\
[X]_{pss-max} &= ([X]_{baseline} + d_X) \cdot f_X .
\end{aligned} \tag{S1}
$$

Afterwards, we computed the minimum difference Δ_X^{min} and maximum difference Δ_X^{max} between the internal concentrations of the glucose baseline and the pseudo-steady state:

$$
\begin{aligned}
\Delta_X^{\mathrm{min}} &= [X]_{pss-min} - ([X]_{baseline} + d_X) \\
\Delta_X^{\mathrm{max}} &= [X]_{pss-max} - ([X]_{baseline} - d_X).
\end{aligned} \tag{S2}
$$

When the differences are negative, Δ_X^{min} and Δ_X^{max} are swapped.

Furthermore, it is important to clarify that not all measured metabolites were used as input in the subsequent runs. Some metabolites are not present in the used metabolic network, especially in the *Escherichia coli* core model. Additionally, the interval defined by Δ_X^{min} and Δ_X^{max} must have a distinct direction meaning that it is not possible that Δ_X^{min} is negative and Δ_X^{max} is positive. Therefore, measured metabolites with a fold change of 1.0 are considered as non-measured metabolites and are assigned the chosen generic ϵ. In some cases, for example if the fold change is close to 1.0 and the baseline deviations are large enough, it is possible that Δ_X^{min} is negative and Δ_X^{max} is positive. In the cases in which the error of the measurement allowed the input deltas to contain both a positive and a negative values and the direction of change was direction was not determinable, the corresponding metabolite was treated as a non-measured metabolite. However, in the cases in which we verified manually that one of the values was closer to zero, we included a zero threshold. Example: Measurement +2 Fold with an error 2.15; The input deltas were set to $[0, 4.15]$ instead of $[-0.15, 4.15]$.

2 TABLES

2.1 Input deltas

Network model	Metabolite Id	$\Delta_X^{\min}$	$\Delta_X^{\max}$
S + L	M_g6p_c	1.790	2.130
S + L	M_f6p_c	0.473	0.547
S + L	M_6pgc_c	0.183	0.237
S + L	M_fdp_c	3.672	3.816
S + L	M_pep_c	-1.060	-0.988
S + L	M_pyr_c	2.632	2.776
S + L	M_cit_c	0.760	0.860
S + L	M_succ_c	7.255	12.605
S + L	M_fum_c	0.880	1.616
S + L	M_mal__L_c	0.488	0.728
S + L	M_adp_c	-0.269	-0.003
S + L	M_gln__L_c	-2.036	-1.492
S + L	M_glc__D_e	-50.000	0
S + L	M_o2_c	-50.000	0
S + L	M_biomass_c	0	50.000
L	M_man6p_c	0.205	0.255
L	M_val__L_c	-0.300	0
L	M_his__L_c	-0.105	-0.015
L	M_phe__L_c	0.106	0.290
L	M_tyr__L_c	-0.084	-0.020
L	M_gly_c	-0.772	-0.228
L	M_trp__L_c	-0.019	-0.007
L	M_pro__L_c	-0.121	0
L	M_asp__L_c	-1.344	-0.672
L	M_asn__L_c	-0.132	0
L	M_thr__L_c	-0.307	0
L	M_ile__L_c	-0.087	0

Table S1. *Calculated variations interval for glucose pulse experiment.* The network model indicates if the metabolite was present in the *E. coli* core model (S) or in the *E. coli* iJO1366 model (L). The interval for each metabolite was calculated based on the baseline concentration and the fold change for the pseudo-steady state taking into account the given small variations for the baseline measurements. To add glucose as a source, $\Delta_{glucose}^{\min}$ was set to -50. Oxygen was added as a source in the same way. Biomass was added as a sink.

Network model	Metabolite Id	$\Delta_X^{\min}$	$\Delta_X^{\max}$
S + L	M_g6p_c	-1.045	-0.915
S + L	M_f6p_c	-0.252	-0.228
S + L	M_6pgc_c	-0.296	-0.275
S + L	M_fdp_c	-0.723	-0.703
S + L	M_pep_c	-1.060	-0.988
S + L	M_cit_c	1.560	1.680
S + L	M_icit_c	1.560	1.680
S + L	M_fum_c	0	0.272
S + L	M_mal__L_c	0.341	0.571
S + L	M_atp_c	-1.180	0
S + L	M_adp_c	-0.269	-0.003
S + L	M_glu__L_c	-4.232	-3.908
S + L	M_gln__L_c	-1.464	-0.888
S + L	M_pyr_c	-50.000	0
S + L	M_o2_c	-50.000	0
S + L	M_biomass_c	0	50.000
L	M_man6p_c	-0.335	-0.309
L	M_ala__L_e	1.986	2.172
L	M_val__L_c	0	0.480
L	M_leu__L_c	0.087	0.249
L	M_phe__L_c	-0.514	-0.410
L	M_tyr__L_c	-0.095	-0.035
L	M_gly_c	-0.556	0
L	M_trp__L_c	-0.012	0
L	M_lys__L_c	-0.240	-0.164
L	M_asp__L_c	-1.806	-1.218
L	M_asn__L_c	-0.184	-0.040
L	M_thr__L_c	-0.394	0

Table S2. *Calculated variations interval for pyruvate pulse experiment.* The network model indicates if the metabolite was present in the *E. coli* core model (S) or in the *E. coli* iJO1366 model (L). The interval for each metabolite was calculated based on the baseline concentration and the fold change for the pseudo-steady state taking into account the given small variations for the baseline measurements. To add pyruvate as a source, $\Delta_{pyruvate}^{\min}$ was set to -50. Oxygen was added as a source in the same way. Biomass was added as a sink.

Network model	Metabolite Id	$\Delta_X^{\min}$	$\Delta_X^{\max}$
S + L	M_g6p_c	-0.505	-0.335
S + L	M_f6p_c	-0.107	-0.073
S + L	M_6pgc_c	-0.252	-0.228
S + L	M_fdp_c	0.051	0.093
S + L	M_pep_c	-0.185	-0.071
S + L	M_pyr_c	0.847	0.921
S + L	M_cit_c	3.16	3.32
S + L	M_fum_c	23.72	33.592
S + L	M_mal__L_c	25.919	27.889
S + L	M_adp_c	-0.269	-0.003
S + L	M_glu__L_c	-4.232	-3.908
S + L	M_succ_c	-50	0
S + L	M_o2_c	-50	0
S + L	M_biomass_c	0	50
L	M_man6p_c	-0.155	-0.121
L	M_val__L_c	-0.35	-0.01
L	M_leu__L_c	-0.102	0
L	M_his__L_c	-0.087	0
L	M_phe__L_c	-0.204	-0.06
L	M_trp__L_c	-0.014	-0.001
L	M_asp__L_c	3.738	5.334
L	M_asn__L_c	0	0.14
L	M_thr__L_c	0.041	0.547

Table S3. *Calculated variations interval for succinate pulse experiment.* The network model indicates if the metabolite was present in the *E. coli* core model (S) or in the *E. coli* iJO1366 model (L). The interval for each metabolite was calculated based on the baseline concentration and the fold change for the pseudo-steady state taking into account the given small variations for the baseline measurements. To add succinate as a source, $\Delta_{succinate}^{\min}$ was set to -50. Oxygen was added as a source in the same way. Biomass was added as a sink. Higher values for sources and sinks were chosen due to larger calculated $\Delta^{\min}$ and $\Delta^{\max}$ than in the other two experiments.

2.2 Input metabolites for random set tests

Metabolite	R_1	R_2	R_3	R_4	R_5
M_cit_c	Incl.	Incl.	Excl.	Incl.	Incl.
M_icit_c	Excl.	Incl.	Incl.	Incl.	Incl.
M_mal__L_c	Incl.	Incl.	Excl.	Excl.	Incl.
M_fum_c	Incl.	Incl.	Incl.	Incl.	Incl.
M_f6p_c	Incl.	Excl.	Incl.	Incl.	Excl.
M_adp_c	Incl.	Incl.	Incl.	Incl.	Excl.
M_6pgc_c	Incl.	Incl.	Incl.	Incl.	Excl.
M_fdp_c	Incl.	Excl.	Incl.	Incl.	Incl.
M_g6p_c	Incl.	Incl.	Incl.	Excl.	Incl.
M_pep_c	Excl.	Incl.	Excl.	Incl.	Excl.
M_atp_c	Incl.	Incl.	Excl.	Excl.	Excl.
M_gln__L_c	Incl.	Incl.	Incl.	Excl.	Incl.
M_glu__L_c	Incl.	Excl.	Excl.	Excl.	Incl.
M_o2_c	Incl.	Excl.	Incl.	Excl.	Excl.
M_pyr_c	Excl.	Incl.	Incl.	Incl.	Incl.

Table S4. *Random metabolites chosen as input, less than 50% excluded.*

Metabolite	R_1	R_2	R_3	R_4	R_5
M_cit_c	Excl.	Excl.	Incl.	Incl.	Excl.
M_icit_c	Excl.	Excl.	Incl.	Incl.	Excl.
M_mal__L_c	Incl.	Incl.	Excl.	Incl.	Excl.
M_fum_c	Incl.	Excl.	Incl.	Incl.	Excl.
M_f6p_c	Excl.	Incl.	Excl.	Excl.	Excl.
M_adp_c	Excl.	Excl.	Excl.	Excl.	Incl.
M_6pgc_c	Excl.	Excl.	Incl.	Excl.	Excl.
M_fdp_c	Incl.	Excl.	Excl.	Excl.	Excl.
M_g6p_c	Incl.	Incl.	Excl.	Excl.	Incl.
M_pep_c	Incl.	Incl.	Excl.	Excl.	Excl.
M_atp_c	Excl.	Excl.	Excl.	Excl.	Excl.
M_gln__L_c	Incl.	Incl.	Excl.	Excl.	Excl.
M_glu__L_c	Excl.	Incl.	Excl.	Excl.	Excl.
M_o2_c	Excl.	Excl.	Excl.	Excl.	Excl.
M_pyr_c	Excl.	Excl.	Incl.	Excl.	Incl.

Table S5. *Random metabolites chosen as input, more than 50% excluded.*

2.3 Results for the *E. coli* iJO1366 model

2.3.1 Pyruvate pulse

Iteration	Objective value	Optimality (%)	Runtime (h)
1	-135.112	5.66	48
2	-134.915	5.48	48
3	-134.879	5.00	29
4	-134.902	6.19	48
5	-134.976	5.00	9
6	-135.309	5.00	23
7	-135.303	6.62	48
8	-135.142	6.31	48
9	-135.117	5.65	48
10	-135.146	6.45	48

Table S6. *Results for pyruvate ($\lambda = 0.1$, $\epsilon = 1.0$).* In total, 10 solutions were computed. The table shows the objective value for each solution and how close this value is to the optimum (in %). The solver stopped either if a solution within 5% of the optimal value was found or after 48 hours. Only in three iterations, the 5% limit was reached. In all other iterations, the solver stopped after 48 hours. However, the obtained solutions had objective values with 7% of the optimum. Thus, we still took them into account when analyzing the predicted active reactions.

2.3.2 Glucose pulse

Iteration	Objective value	Optimality (%)	Runtime (h)
1	-147.134	8.39	48
2	-147.153	8.35	48
3	-147.744	6.58	48
4	-147.75	8.09	48
5	-147.744	7.72	48
6	-147.772	7.47	48
7	-147.809	8.32	48
8	-147.998	7.30	48
9	-148.259	7.47	48
10	-148.331	8.08	48

Table S7. *Results for glucose ($\lambda = 0.1$, $\epsilon = 1.0$).* In total, 10 solutions were computed. The table shows the objective value for each solution and how close this value is to the optimum (in %). The solver stopped either if a solution within 5% of the optimal value was found or after 48 hours. The 5% limit was never reached and the solver always stopped after 48 hours. All computed solutions were within 8.50% of the optimal value.

2.4 Figures

2.4.1 Active reactions in the *E. coli* core model

The following figures show the results for the three pulse experiments for different parameters. The metabolites that were given as input are highlighted in blue if the corresponding input deltas were below zero and red if they were above zero. Reactions that are highlighted in orange were chosen in almost all of the enumerated solutions. Reactions that are yellow were chosen only in around half of the solutions. White reactions were not chosen in any solution.

2.5 Pyruvate pulse

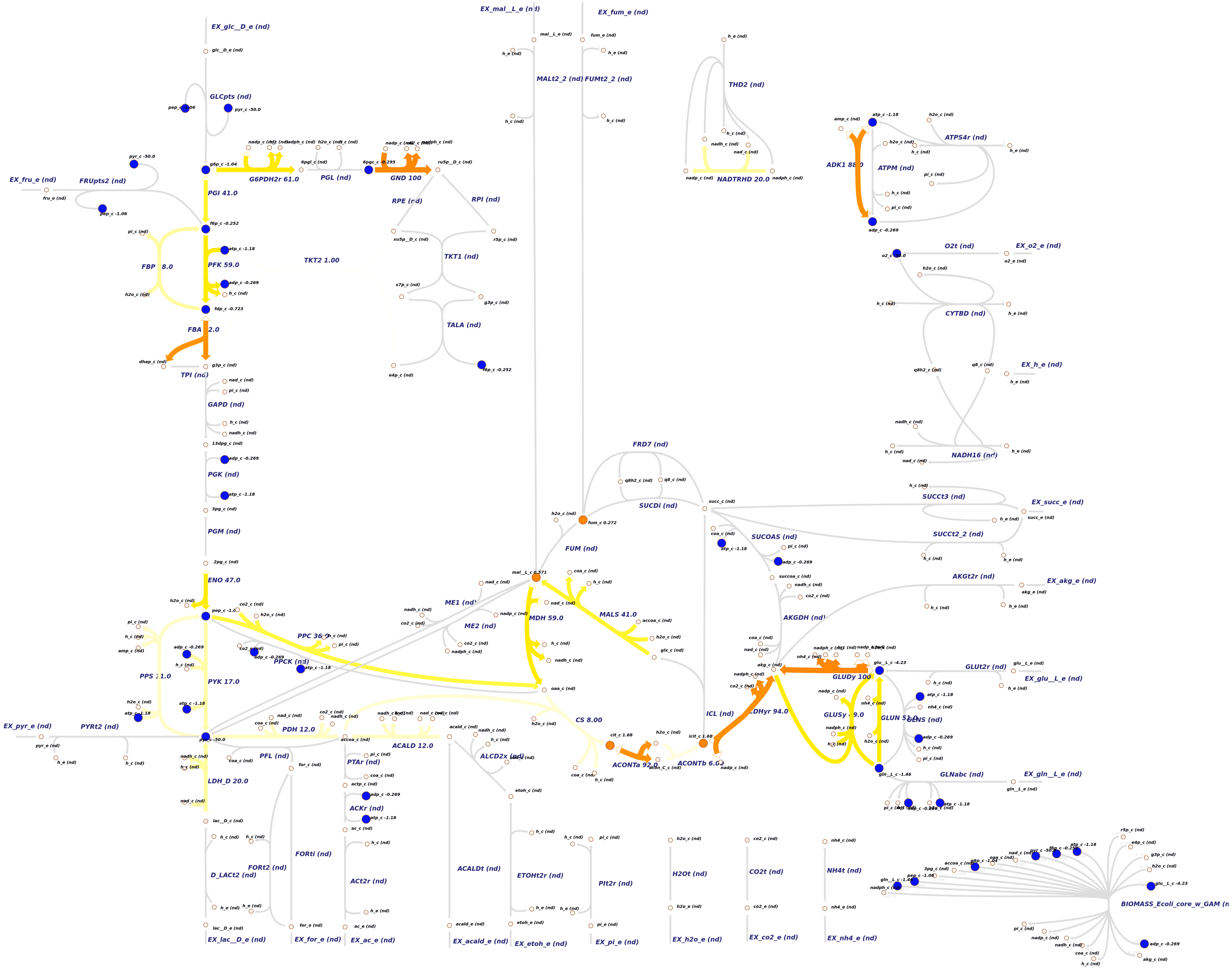

Figure S1. *E. coli core model - Results for pyruvate pulse ($\lambda = 0.9$, $\epsilon = 10$).* The active reactions are disconnected. Since $\lambda = 0.9$, the optimization prioritizes the minimization of the number of active reactions, fewer active reactions are thus chosen in total and the accumulation/depletion of unmeasured metabolites is higher. The figure was created using *Escher* King et al. (2015).

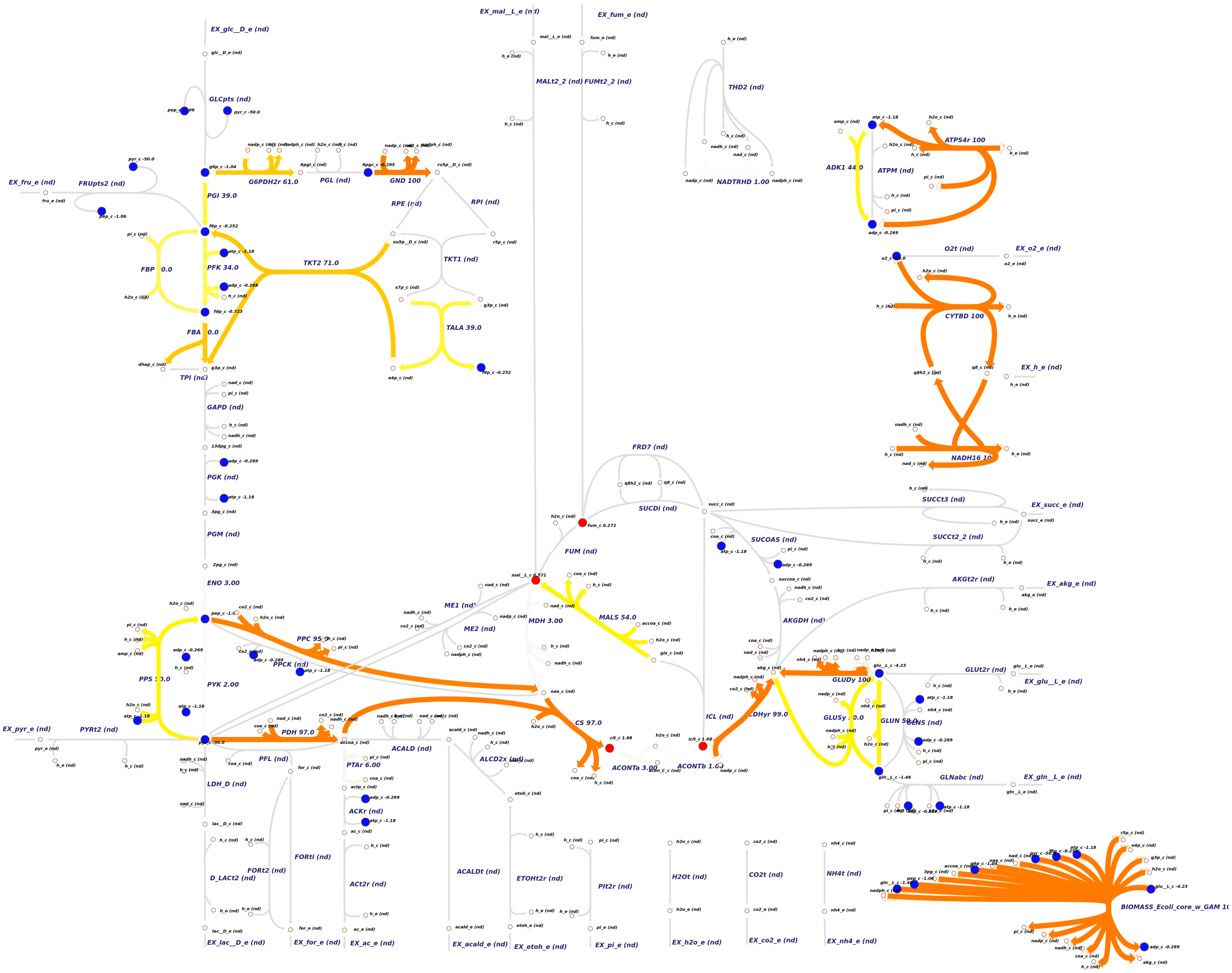

Figure S2. *E. coli core model - Results for pyruvate pulse* ($\lambda = 0.9$, $\epsilon = 5$). Similar reactions are active as for $\lambda = 0.9$ and $\epsilon = 10$. An important difference is that the biomass reaction is part of the solution. The figure was created using *Escher* King et al. (2015).

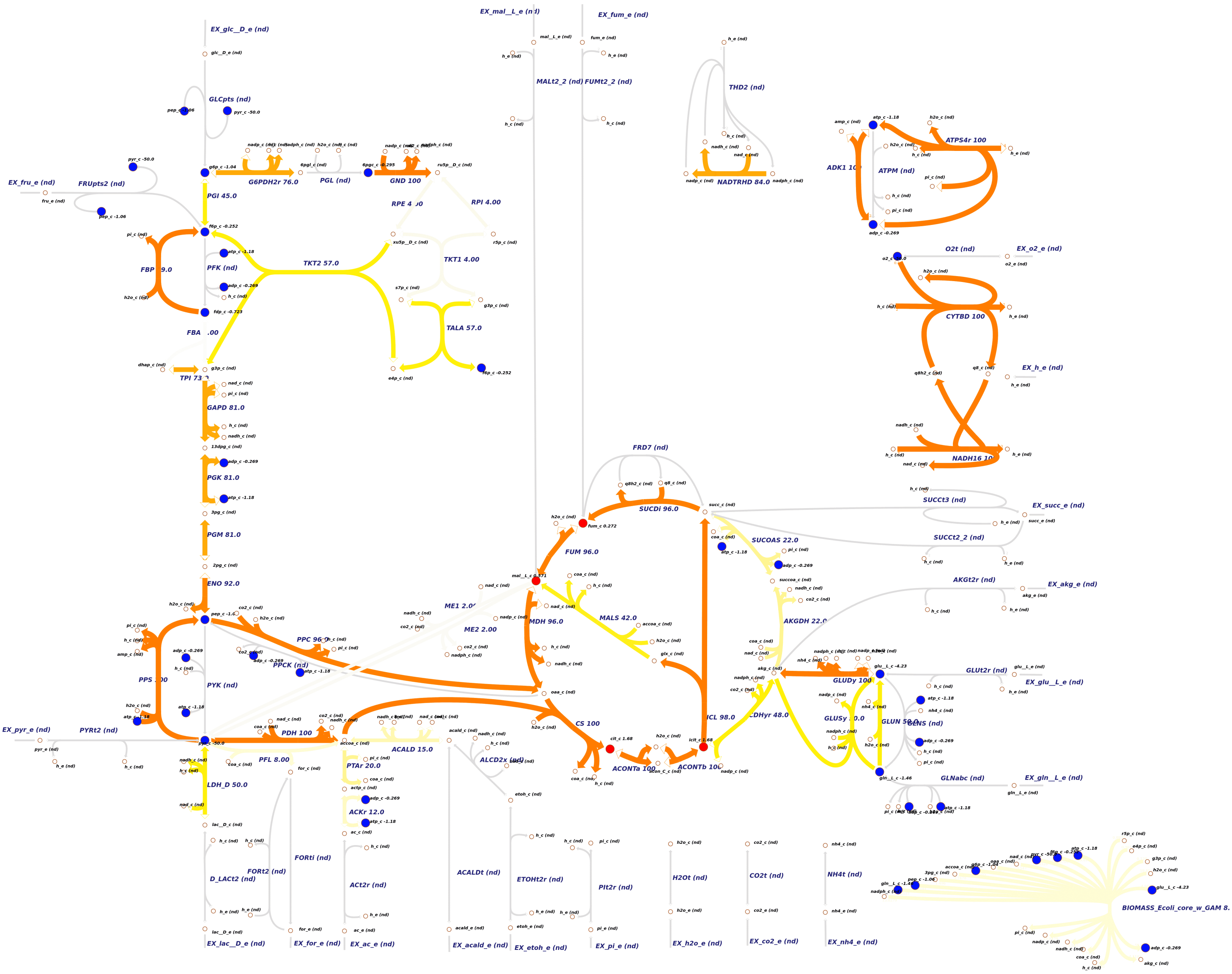

Figure S3. *E. coli core model - Results for pyruvate pulse ($\lambda = 0.5$, $\epsilon = 10$).* After lowering λ to 0.5, the solutions already contain more active reactions and we are able to see connected pathways that are active during the metabolic shift. The biomass reaction is only chosen in 8 out of the 100 solutions. The figure was created using *Escher* King et al. (2015).

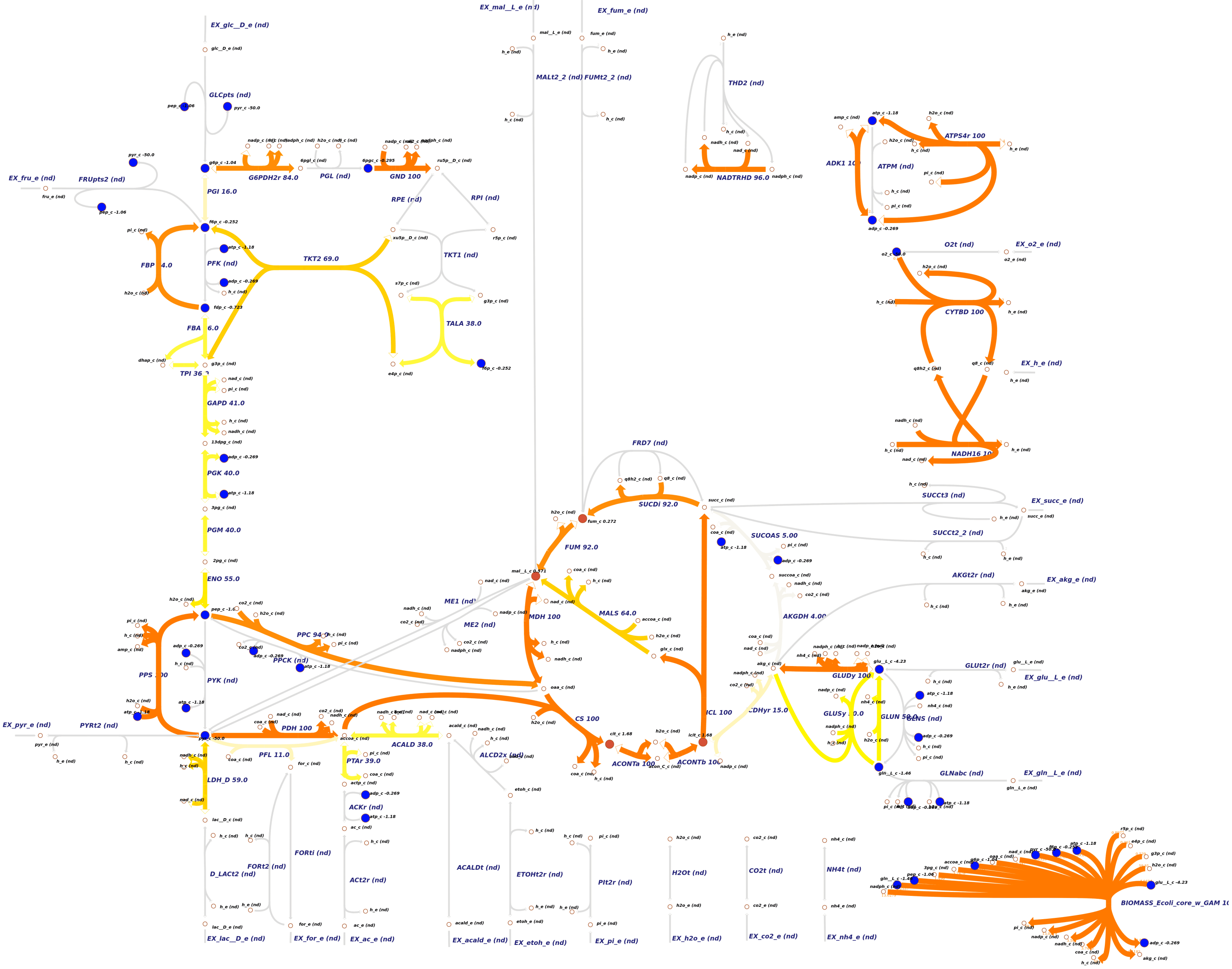

Figure S4. *E. coli core model - Results for pyruvate pulse ($\lambda = 0.5$, $\epsilon = 5$).* The results are similar to $\lambda = 0.5$ and $\epsilon = 10$. However, again the biomass reaction is active in all solutions. The figure was created using *Escher* King et al. (2015).

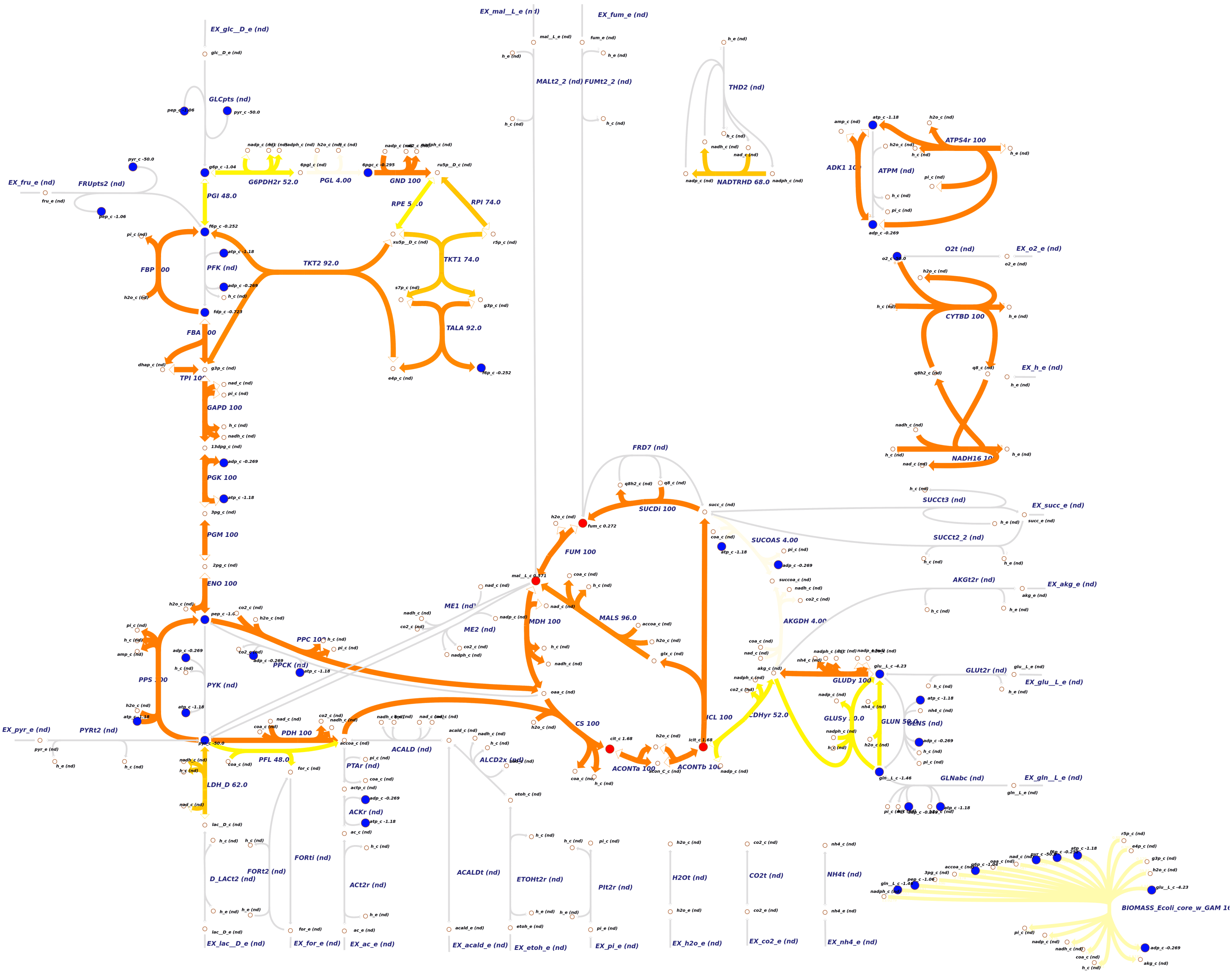

Figure S5. *E. coli core model - Results for pyruvate pulse* ($\lambda = 0.1$, $\epsilon = 10$)*.* We can see connected pathways. The expected reactions of the gluconeogenesis and part of the TCA cycle are active in all 100 solutions. The reversible reactions of the gluconeogenesis are chosen in the correct direction. The figure was created using *Escher* King et al. (2015).

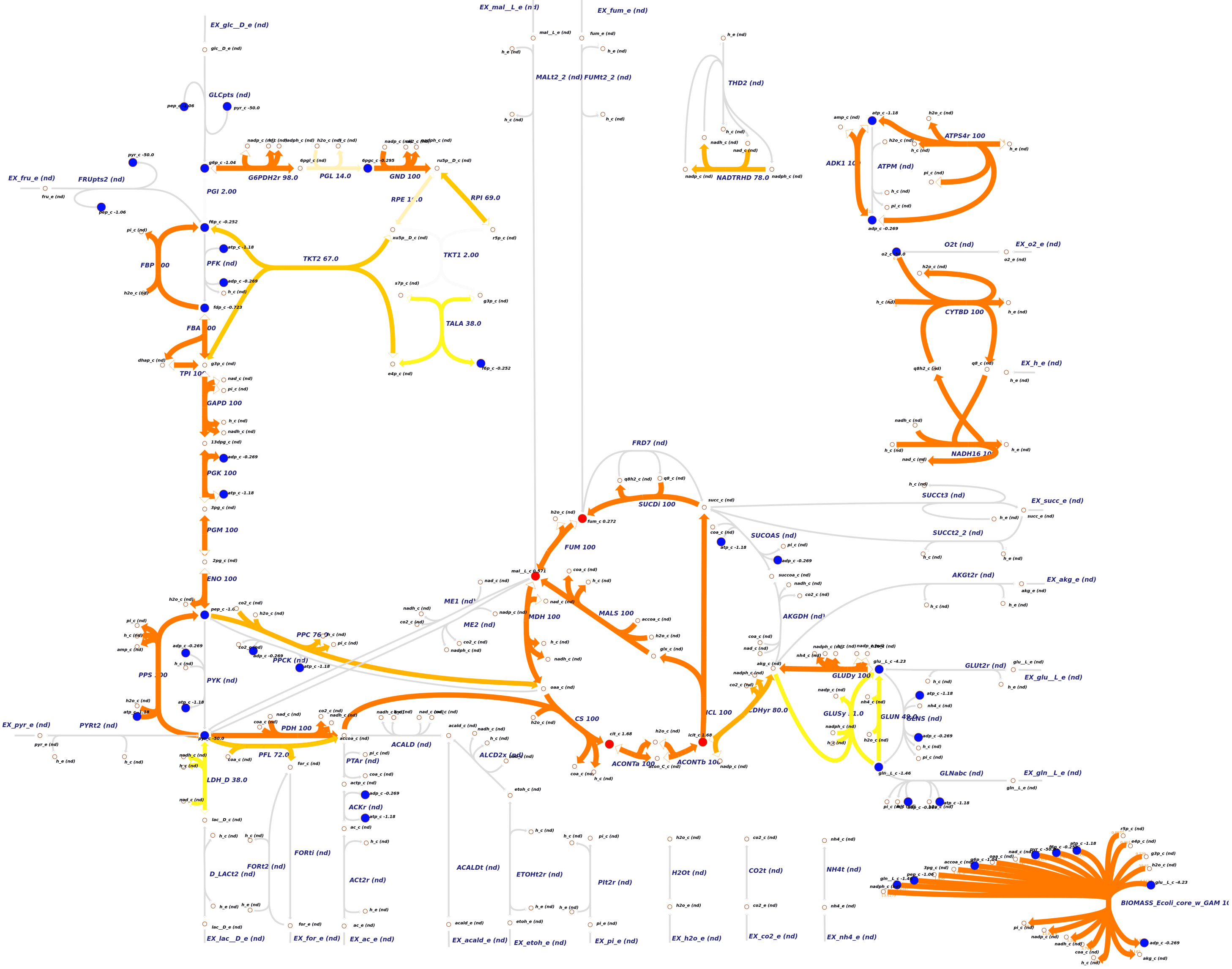

Figure S6. *E. coli core model - Results for pyruvate pulse* ($\lambda = 0.1$, $\epsilon = 5$). The results are similar to $\lambda = 0.1$ and $\epsilon = 10$. However, like for the higher λ, $\epsilon = 5$ results in the biomass reaction getting chosen in all 100 solutions. The figure was created using *Escher* King et al. (2015).

2.6 Glucose pulse

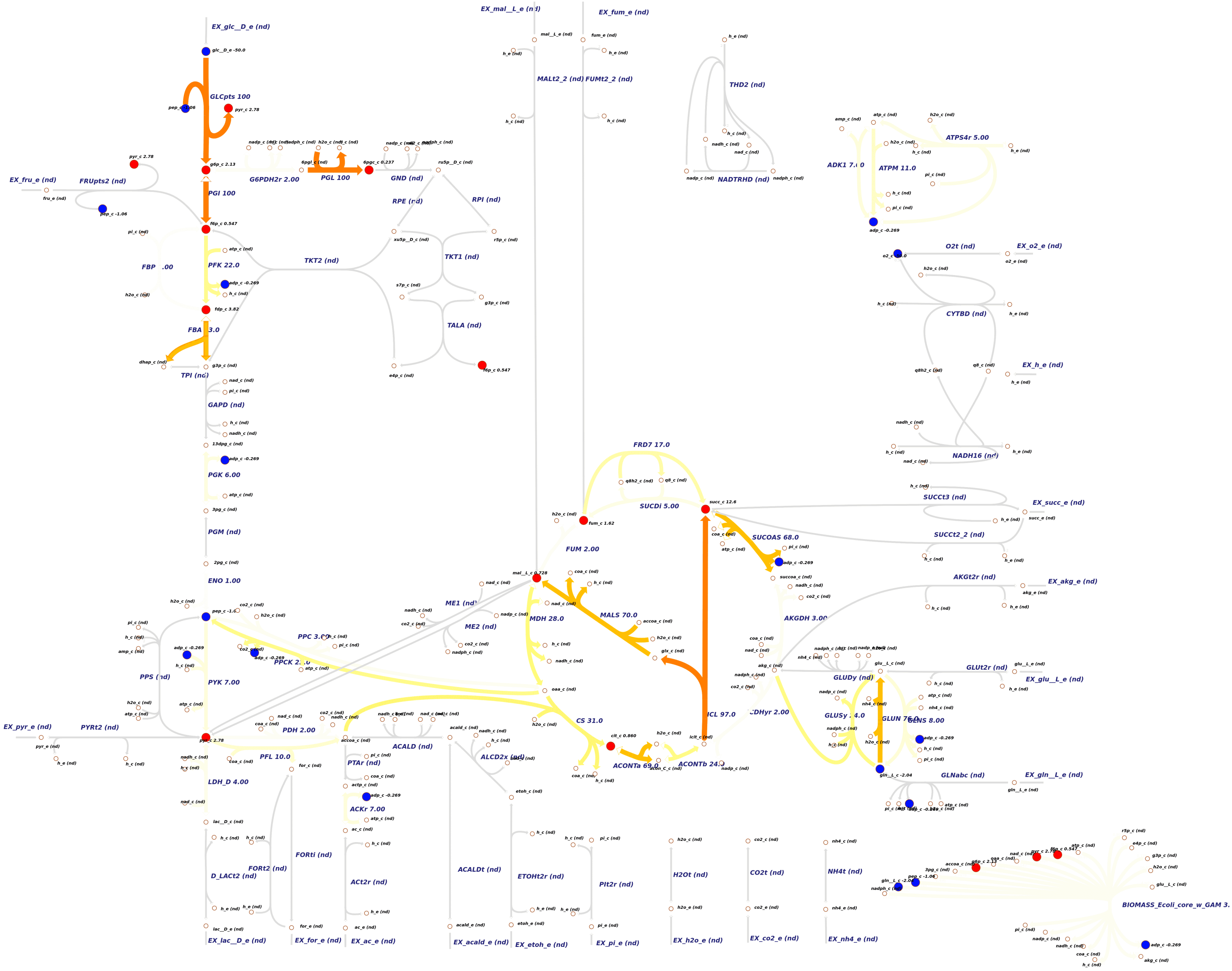

Figure S7. *E. coli core model - Results for glucose pulse* ($\lambda = 0.9$, $\epsilon = 5$). Similar to the results of the pyruvate pulse for $\lambda = 0.9$, the active reactions in the solutions are disconnected. The figure was created using *Escher* King et al. (2015).

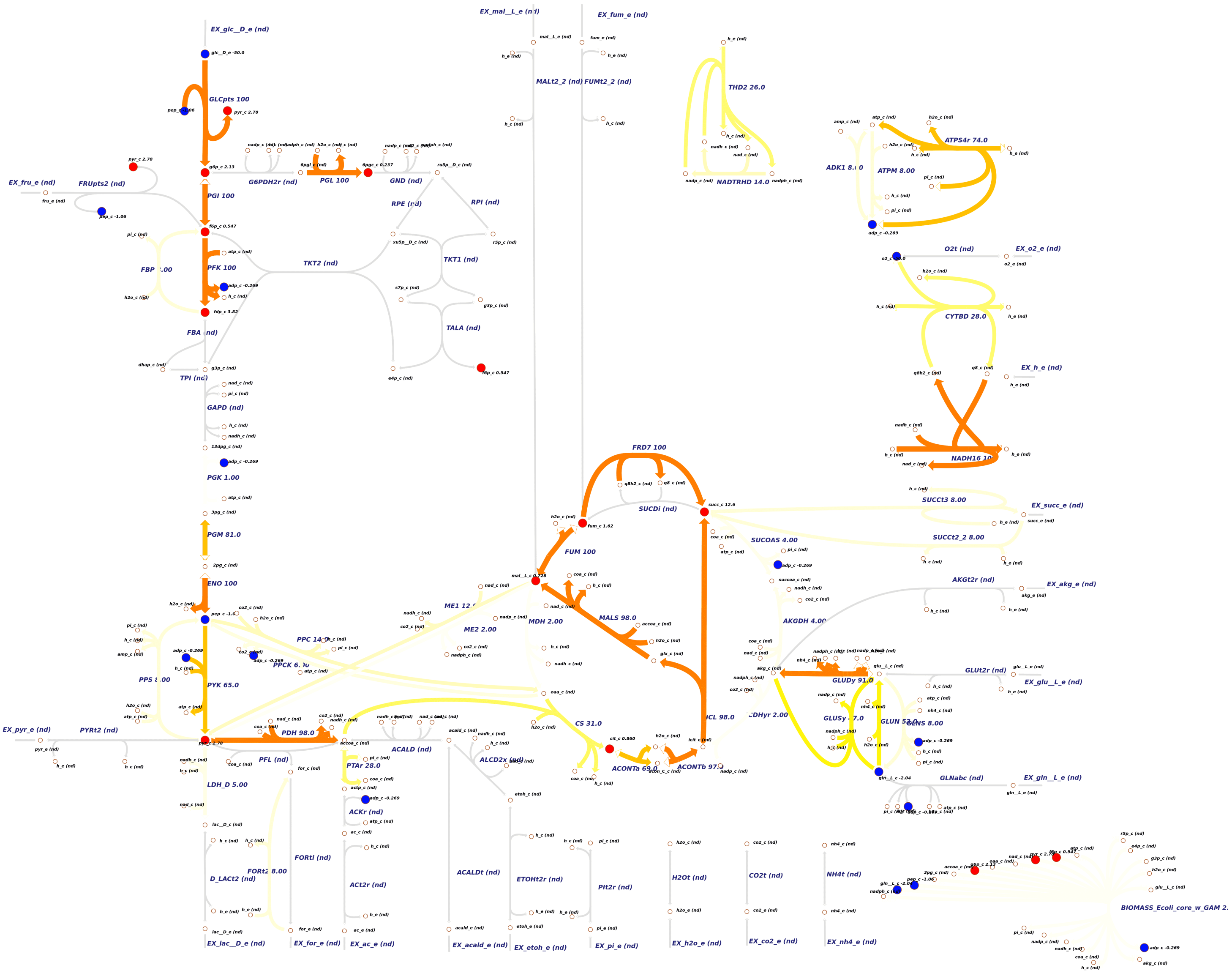

Figure S8. *E. coli core model - Results for glucose pulse* ($\lambda = 0.5$, $\epsilon = 5$). The result is still similar to $\lambda = 0.9$ although more reactions get chosen more frequently in the different solutions. The figure was created using *Escher* King et al. (2015).

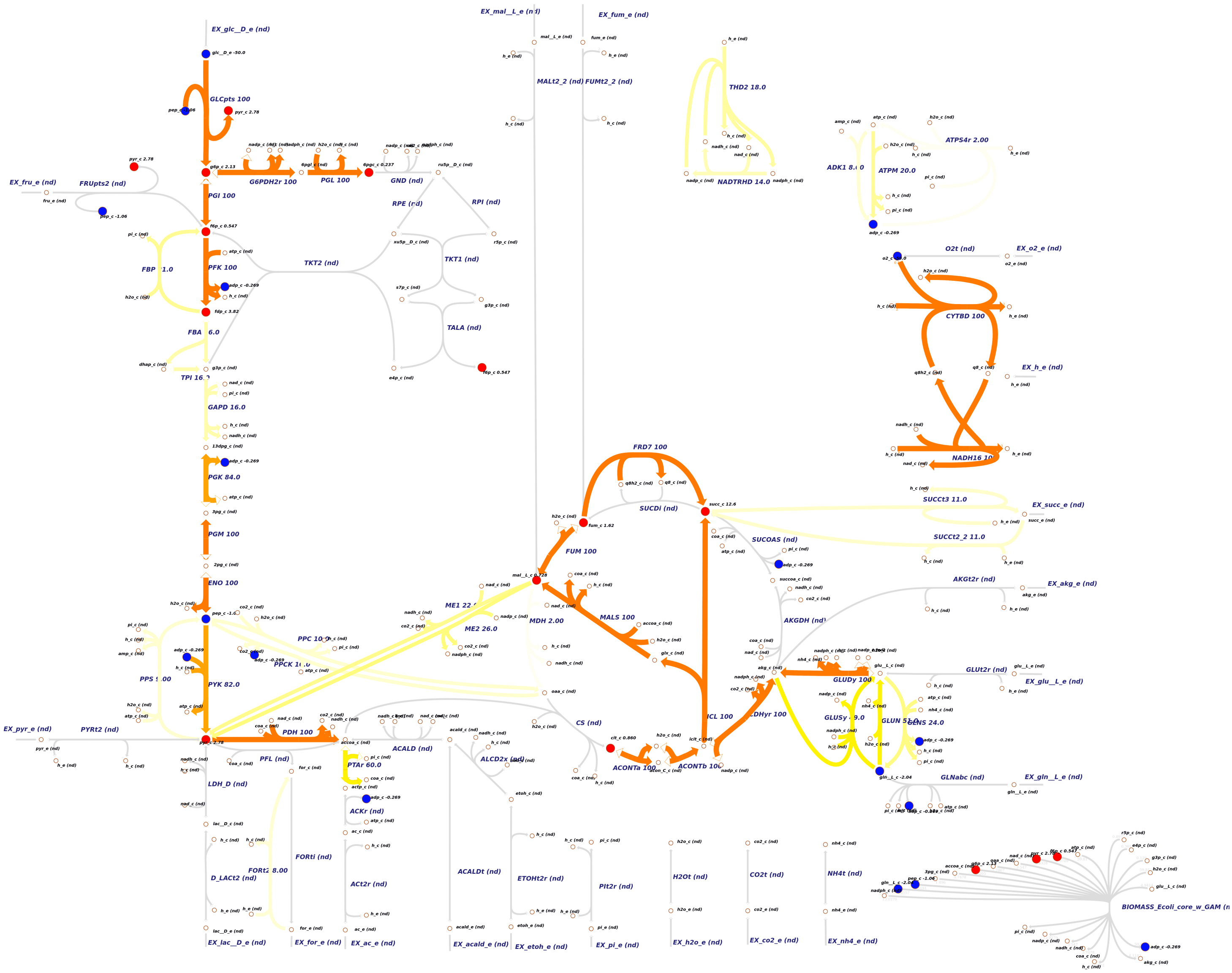

Figure S9. *E. coli core model - Results for glucose pulse* ($\lambda = 0.1$, $\epsilon = 5$). Even for $\lambda = 0.1$, we are not able to see that the expected reactions of the glycolysis and parts of the TCA cycle are active in most of the solutions. Therefore, λ was decreased further to see if it is possible to improve the results and to obtain connected pathways. The figure was created using *Escher* King et al. (2015).

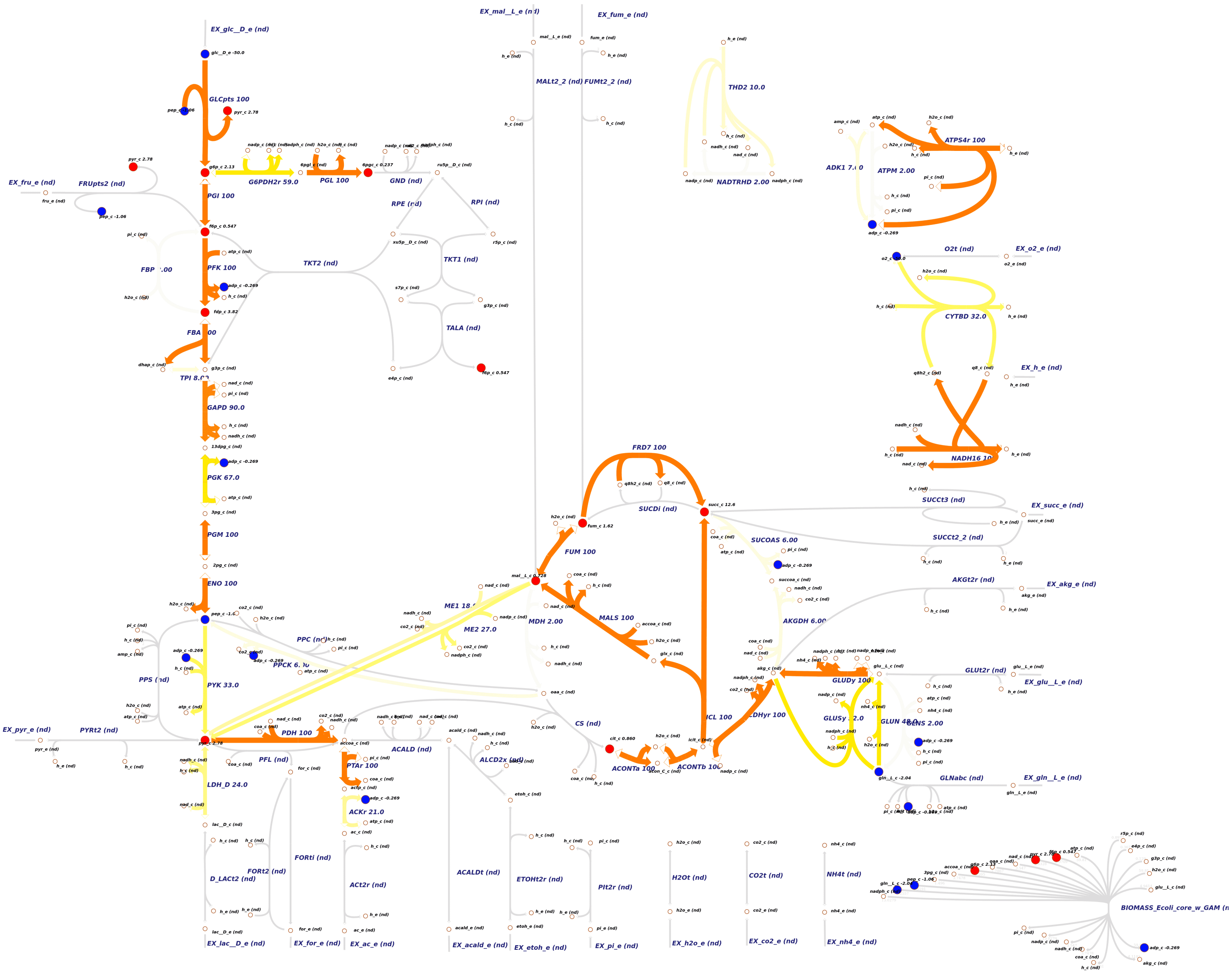

Figure S10. *E. coli core model - Results for glucose pulse ($\lambda = 0.1$, $\epsilon = 2$).* After decreasing λ to 2, more reactions of the glycolysis were active more frequently in the 100 solutions. The figure was created using *Escher* King et al. (2015).

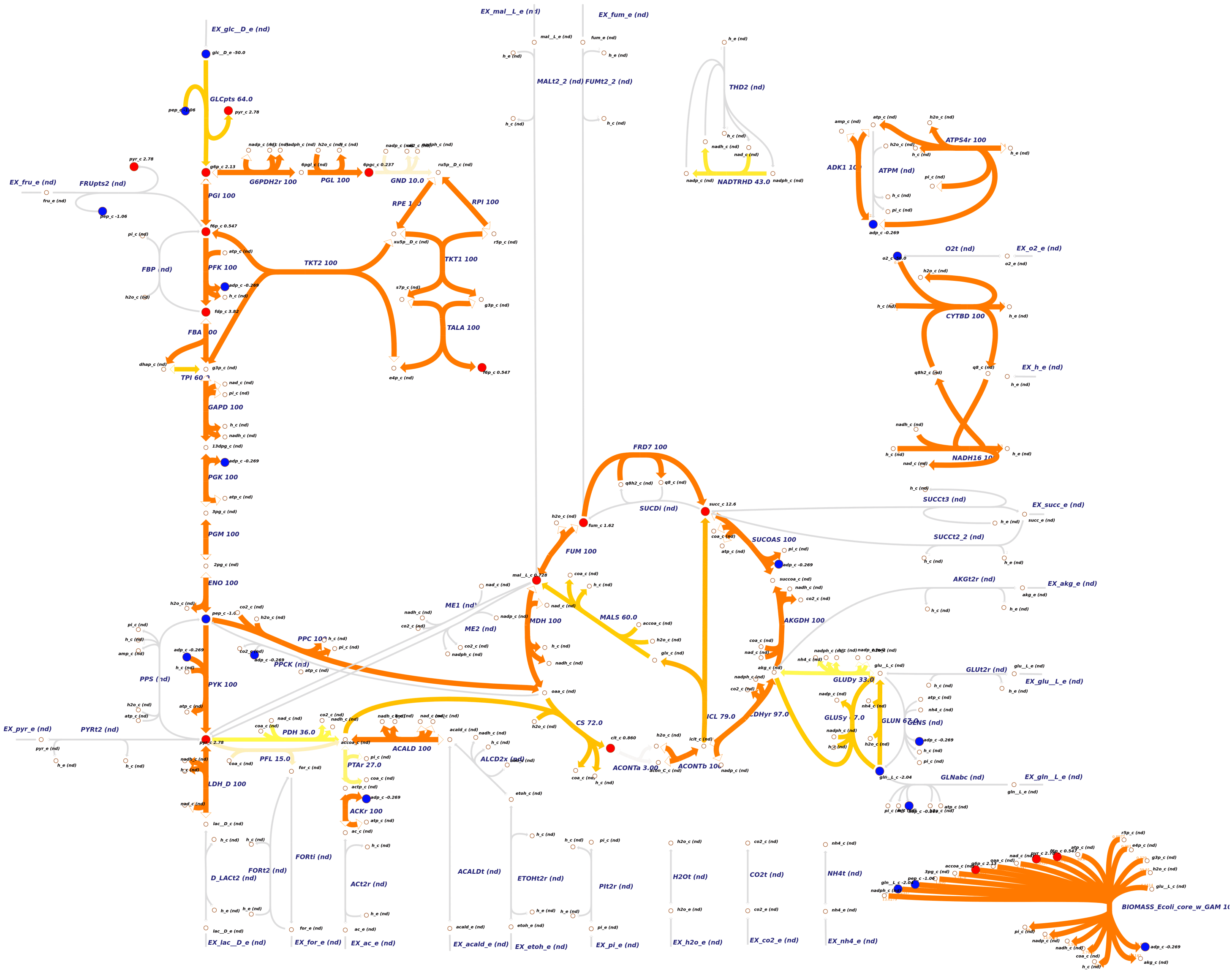

Figure S11. *E. coli core model - Results for glucose pulse ($\lambda = 0.1$, $\epsilon = 1.2$).* The previous result could be improved even further by decreasing λ to 1.2. Reactions of the glycolysis and the TCA cycle are active in all 100 solutions. The active reactions of the glycolysis pathway that are reversible are chosen in the correct direction. Furthermore, in contrast to the previous results, the biomass reaction is part of the solution. However, the reaction that transports glucose is only active in 64 out of 100 solutions. Lowering λ to 1.1 rendered the optimization problem infeasible. The figure was created using *Escher* King et al. (2015).

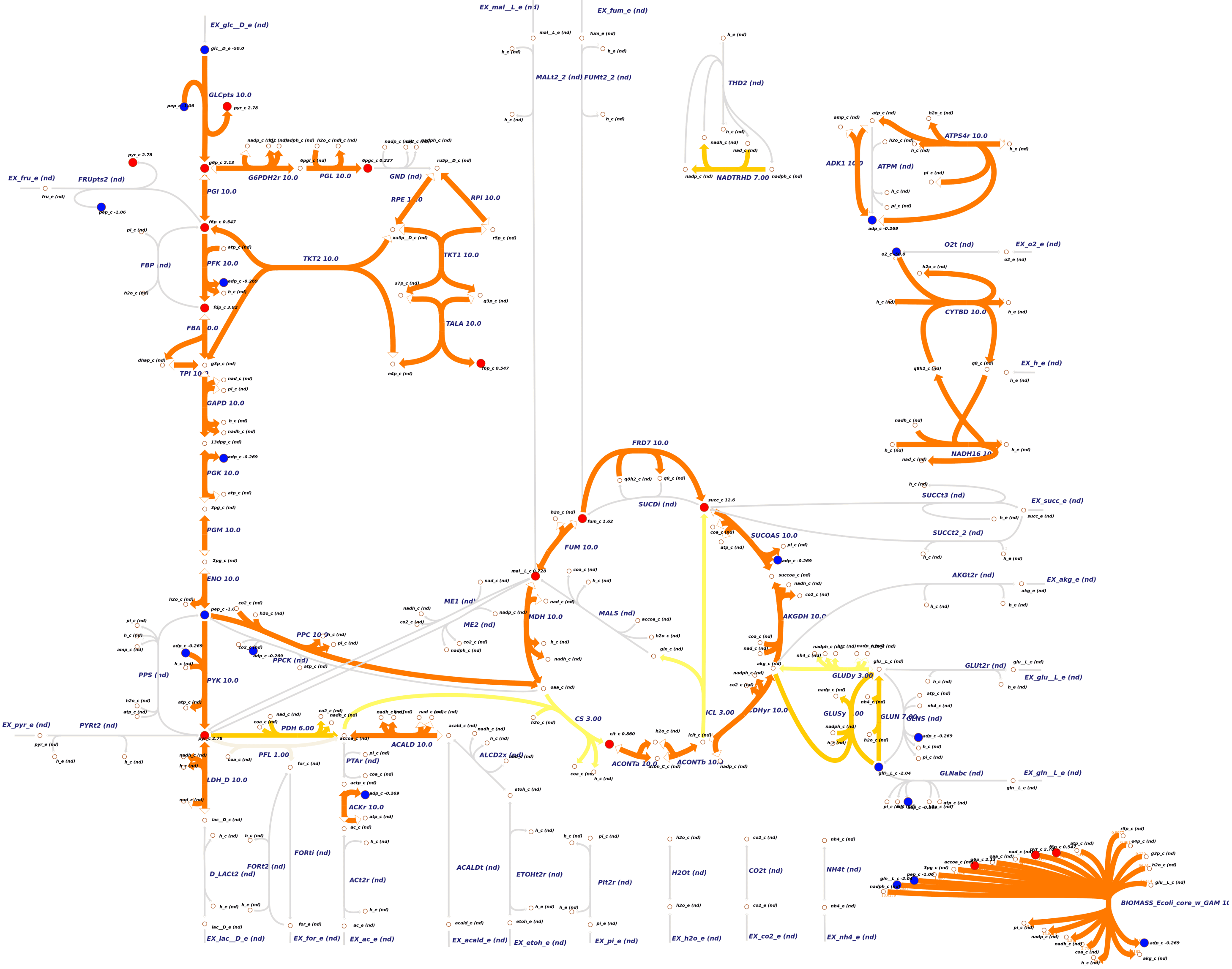

Figure S12. *E. coli core model - First ten solutions for glucose pulse* (*$\lambda = 0.1$, $\epsilon = 1.2$*). In the first ten solutions, the transport reaction for glucose is always active. The other active reactions are very similar to the results for 100 solutions. The figure was created using *Escher* King et al. (2015).

2.7 Succinate pulse

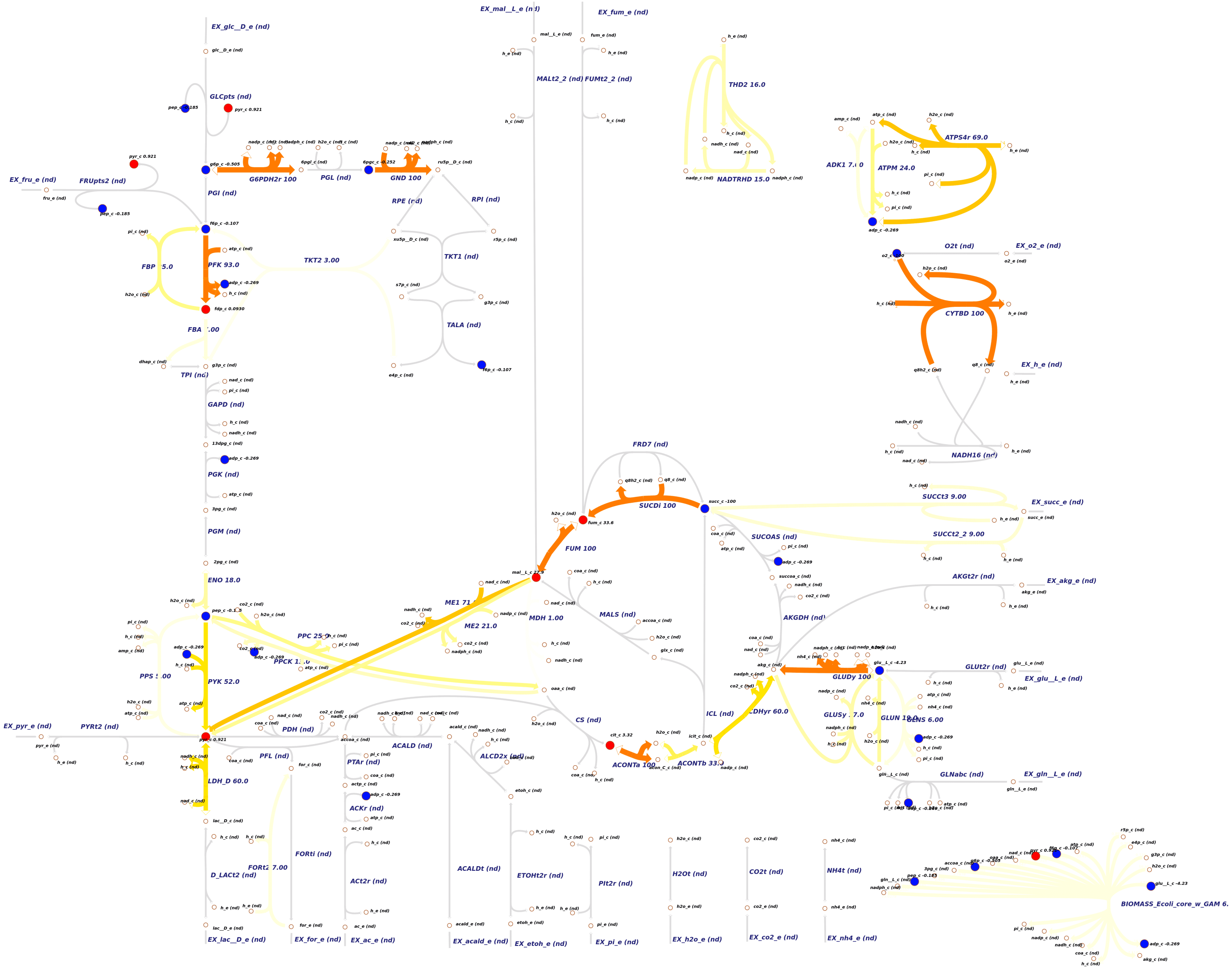

Figure S13. *E. coli core model - Results for succinate pulse (*$\lambda = 0.9$*,* $\epsilon = 10$*).* We can see similarities to the results of the other pulse experiments. For $\lambda = 0.9$ the active reactions are disconnected. The figure was created using *Escher* King et al. (2015).

REFERENCES

King, Z. A., Dräger, A., Ebrahim, A., Sonnenschein, N., Lewis, N. E., and Palsson, B. O. (2015). Escher: a web application for building, sharing, and embedding data-rich visualizations of biological pathways. *PLoS Comput Biol* 11, e1004321

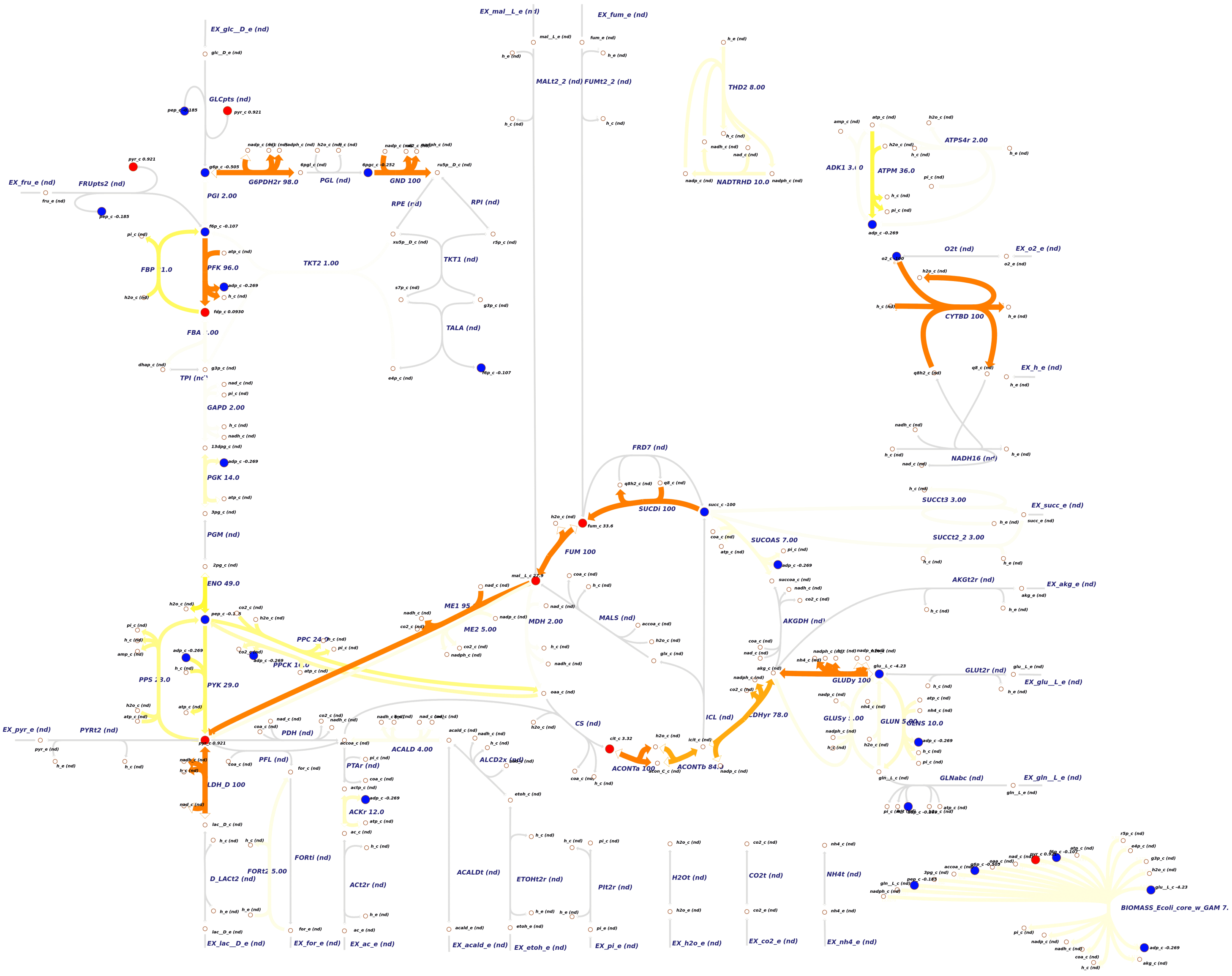

Figure S14. *E. coli core model - Results for succinate pulse ($\lambda = 0.9$, $\epsilon = 5$).* For $\lambda = 0.9$ the active reactions are disconnected. The figure was created using *Escher* King et al. (2015).

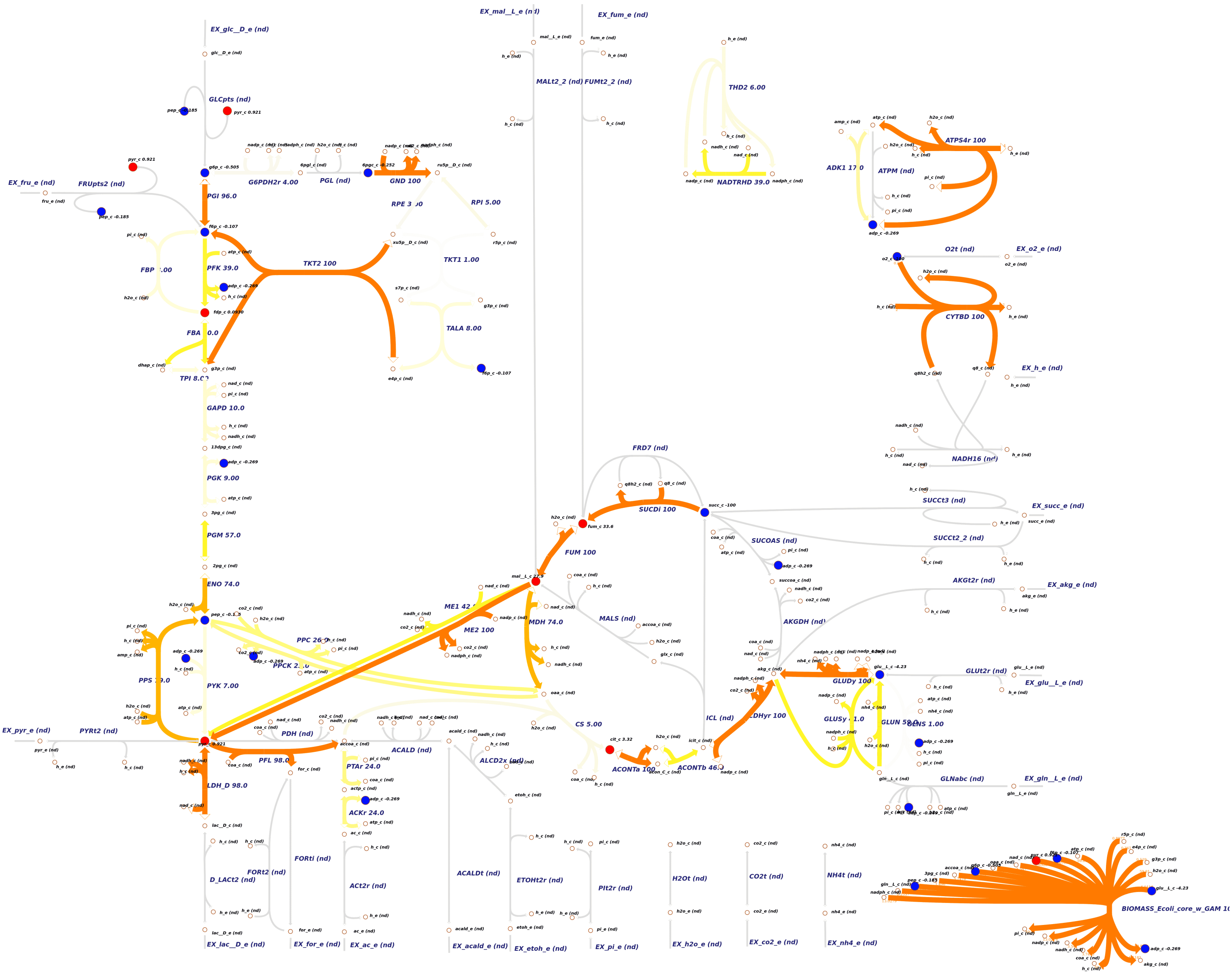

Figure S15. *E. coli core model - Results for succinate pulse* ($\lambda = 0.5$, $\epsilon = 10$). The results are already more connected than for $\lambda = 0.5$ but we are not able to obtain the expected gluconeogenesis pathway. The figure was created using *Escher* King et al. (2015).

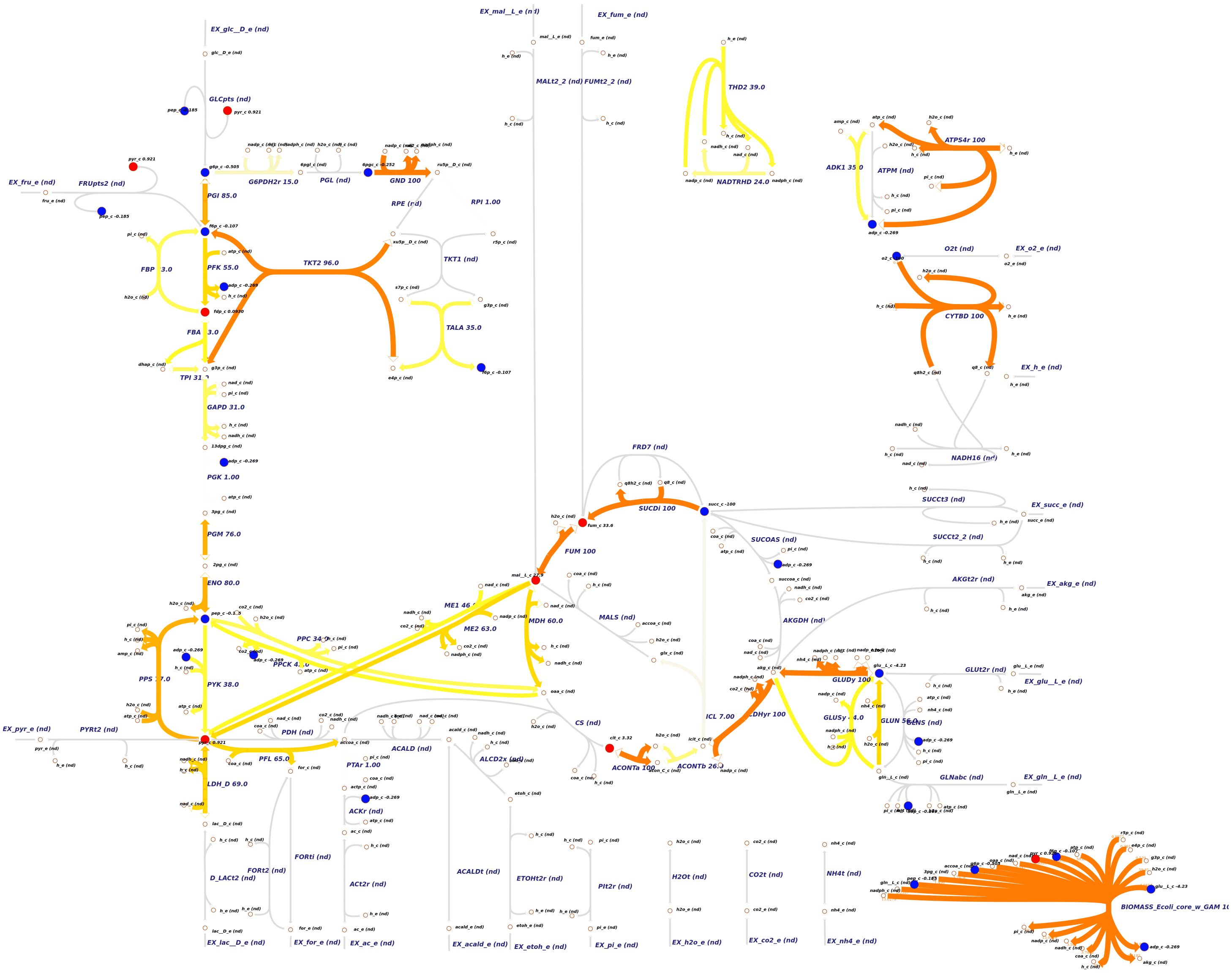

Figure S16. *E. coli core model - Results for succinate pulse* ($\lambda = 0.5$, $\epsilon = 5$). The results are already more connected than for $\lambda = 0.5$ but we are not able to obtain the expected gluconeogenesis pathway. The figure was created using *Escher* King et al. (2015).

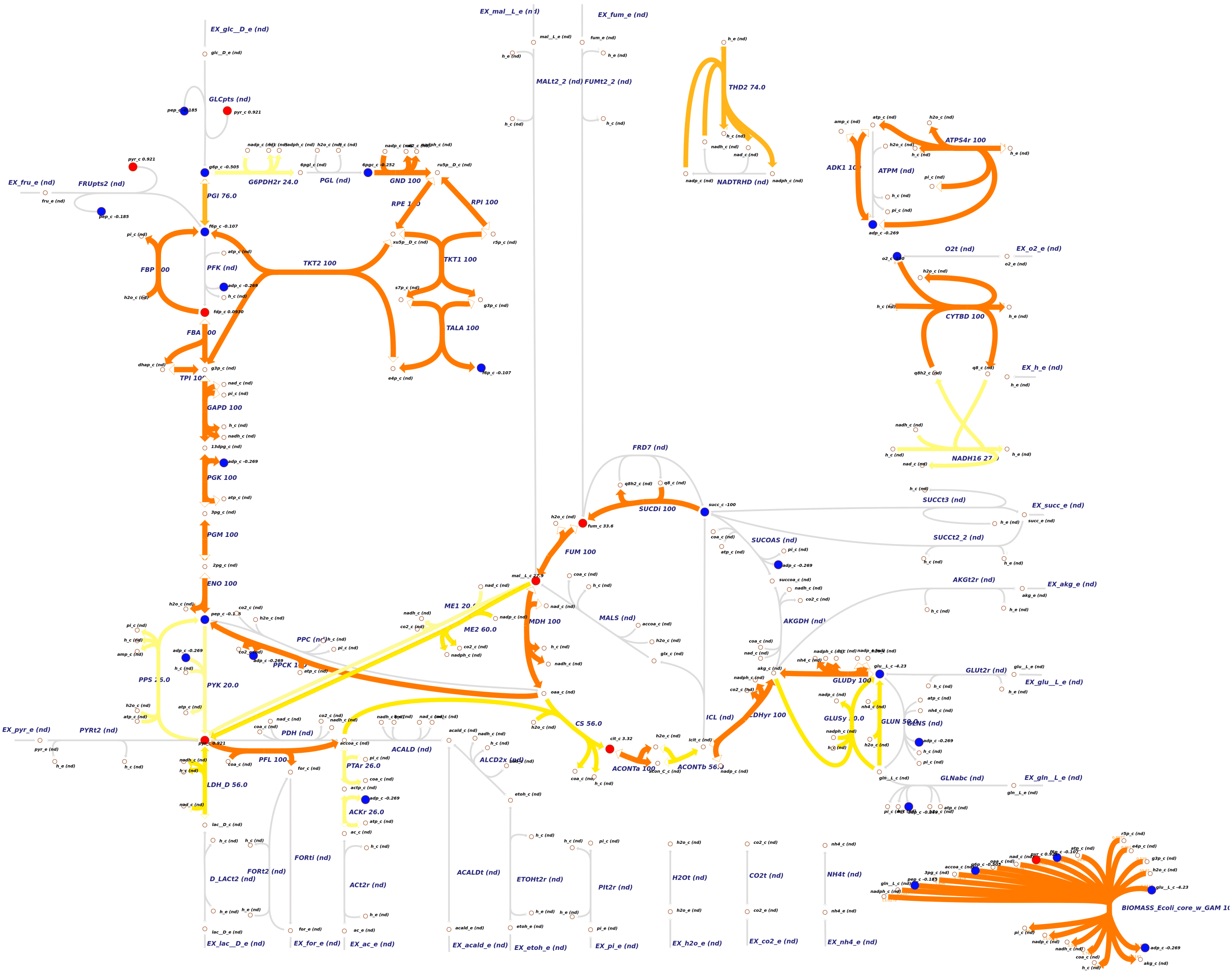

Figure S17. *E. coli core model - Results for succinate pulse* ($\lambda = 0.1$, $\epsilon = 10$). The reactions of the gluconeogenesis pathway and the reactions that transform succinate in the TCA cycle and subsequently into pyruvate are active in all 100 solutions. The figure was created using *Escher* King et al. (2015).

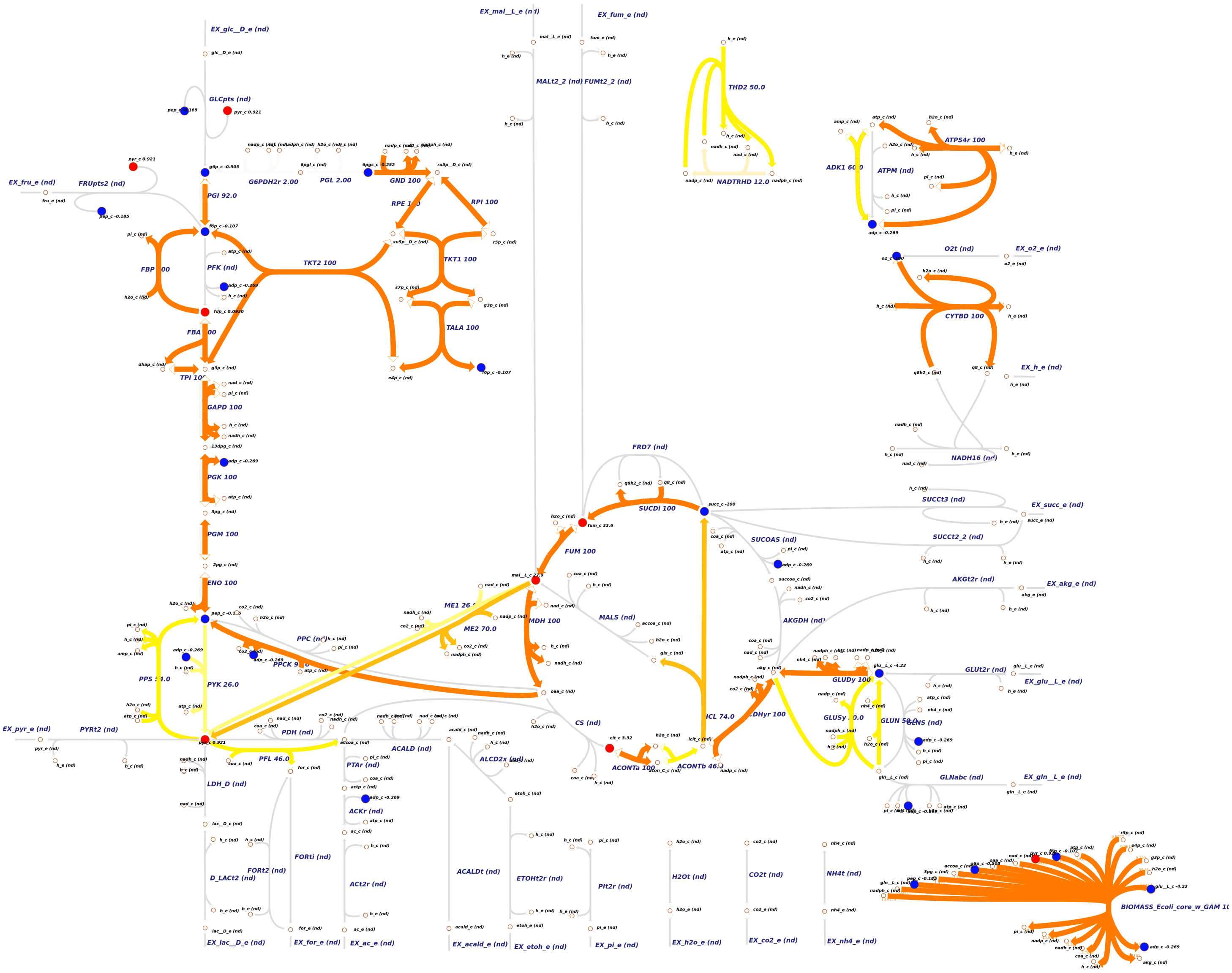

Figure S18. *E. coli core model - Results for succinate pulse ($\lambda = 0.1$, $\epsilon = 5$).* The result is similar to the results for $\lambda = 0.1$ and $\epsilon = 10$. The figure was created using *Escher* King et al. (2015).
